# Supplementary material for: Screening of Hydrocarbon-Stapled Peptides for Inhibition of Calcium-Triggered Exocytosis
Source: Front Pharmacol. 2022 Jun 17;13:891041. doi: 10.3389/fphar.2022.891041 (PMC9258623; doi:10.3389/fphar.2022.891041)

new england peptide™

Certificate of Analysis

**Sequence:** [Cyc(5,9;15,19)]Ac-SKDA(S5)IRT(S5)VMLDE(S5)GEQ(S5)DR-amide

**Peptide Name:**	**Date:** 8/8/2017

**Order#:** P611359	**Lot#:** LB1540	**Amount:** 5.0mg

Quality Control Specifications:

QC Test	QC Specifications	Results
Purity by HPLC	**≥90%** by percent area	**Pass**
Mass Identification by Mass Spectral Analysis	Calculated Mass within 0.1% of Molecular Weight: **2506**	**Pass**
Concentration/ Net Peptide	Amino Acid Analysis (AAA) determining original concentration/net peptide content.	**N/A**

Product: Research Grade Custom Peptide containing traces of Trifluoroacetate (TFA) salts.

Formulation:
Final concentration: N/A
Final form: Dry

Stability and Conditions: Refer to the Quality Control Detail Information on our website at www.newenglandpeptide.com/support/quality-control-information . As always, NEP has individual batch records stored electronically for each peptide that includes traceable lot numbers of raw materials used during synthesis. Should you require this information, email sales@newenglandpeptide.com with your peptide lot number.

Notes (if applicable):

JM
Approval/Initials

For Science... From Science.

New England Peptide Inc., 65 Zub Lane, Gardner, MA 01440 ▪ **Phone** 888-343-5974 ▪ **Fax** 978-630-0021

www.NewEnglandPeptide.com

Peptide QC Report LB1540R DRY

Analysis Name	D:\Data\LB1540R DRY_143504_P1-B-3_01_77106.D
Sample Name	LB1540R DRY
Method	APRIL20171.2mLperMIN_NEPOAHIGH_77106.m
Instrument	amaZon SL

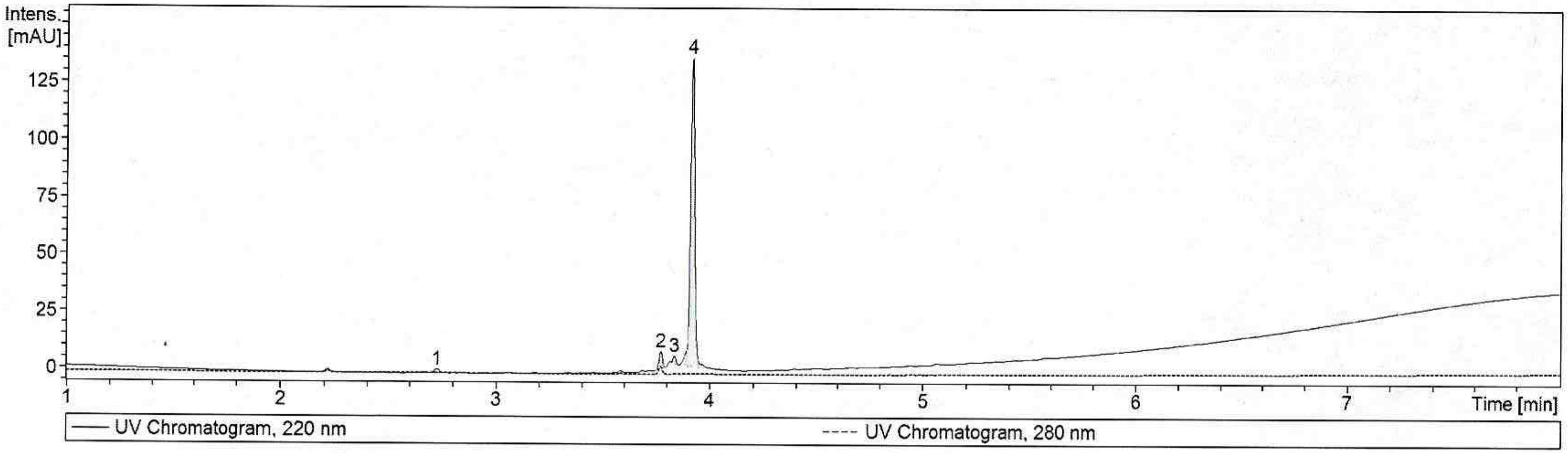

Target Mass	Meas. Mass	Expec. Mass	Delt. Mr [Da]	Intensity	Area	Area Fraction [%]
Cmpd 4; 3.92 min; Pep Mr: 2505.27	2505.27	2506.00	-0.73	136	177	92.8

#	RT [min]	Area	Area Frac. %
1	2.72	2.8133	1.47
2	3.77	7.9101	4.14
3	3.84	2.9568	1.55
4	3.92	177.3334	92.84

new england peptide

Peptide QC Report LB1540R DRY

Cmpd 4; 3.92 min; Pep Mr: 2505.27

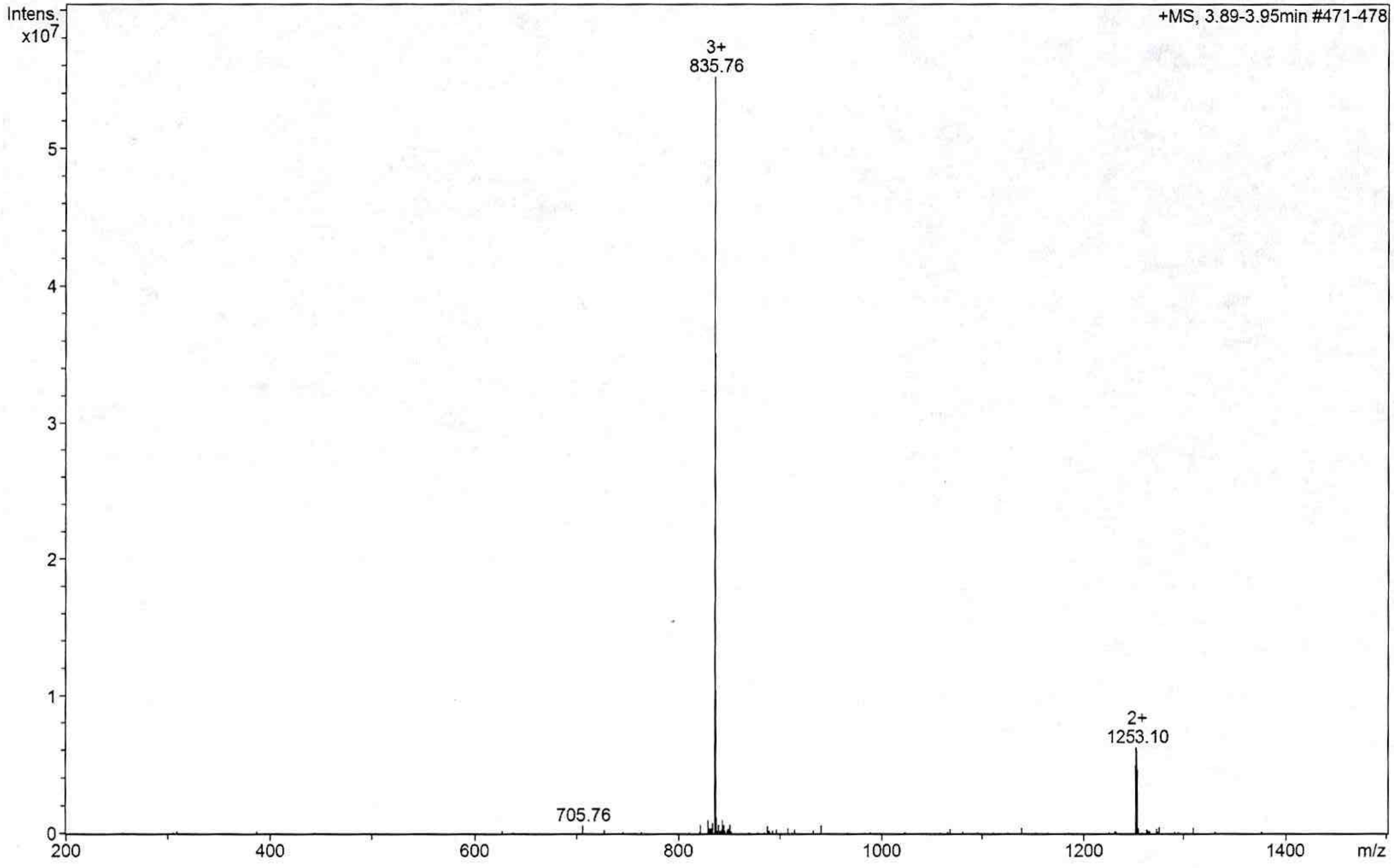

Supplement: Supplementary file 1 [file DataSheet7.PDF]
