# Supplementary material for: Screening of Hydrocarbon-Stapled Peptides for Inhibition of Calcium-Triggered Exocytosis
Source: Front Pharmacol. 2022 Jun 17;13:891041. doi: 10.3389/fphar.2022.891041 (PMC9258623; doi:10.3389/fphar.2022.891041)

new england peptide™

Certificate of Analysis

**Sequence:** [Cyc(5,12)]Ac-SKDA(R8)IRTLVM(S5)DEQGEQL-amide

**Peptide Name:**	**Date:** 8/8/2017

**Order#:** P611359	**Lot#:** LB1503	**Amount:** 5.3mg

Quality Control Specifications:

QC Test	QC Specifications	Results
Purity by HPLC	≥**90%** by percent area	**Pass**
Mass Identification by Mass Spectral Analysis	Calculated Mass within 0.1% of Molecular Weight: **2267**	**Pass**
Concentration/ Net Peptide	Amino Acid Analysis (AAA) determining original concentration/net peptide content.	**N/A**

Product: Research Grade Custom Peptide containing traces of Trifluoroacetate (TFA) salts.

Formulation:
Final concentration: N/A
Final form: Dry

Stability and Conditions: Refer to the Quality Control Detail Information on our website at www.newenglandpeptide.com/support/quality-control-information . As always, NEP has individual batch records stored electronically for each peptide that includes traceable lot numbers of raw materials used during synthesis. Should you require this information, email sales@newenglandpeptide.com with your peptide lot number.

Notes (if applicable): Both isomers will be counted towards final purity.

Approval/Initials

For Science... From Science.

New England Peptide Inc., 65 Zub Lane, Gardner, MA 01440 ■ **Phone** 888-343-5974 ■ **Fax** 978-630-0021

www.NewEnglandPeptide.com

Peptide QC Report LB1503 33-41

Analysis Name	D:\Data\LB1503 33-41_143121_P1-E-8_01_76849.d
Sample Name	LB1503 33-41
Method	APRIL20171.2mLperMIN_NEPOAHIGH_76849.m
Instrument	amaZon SL

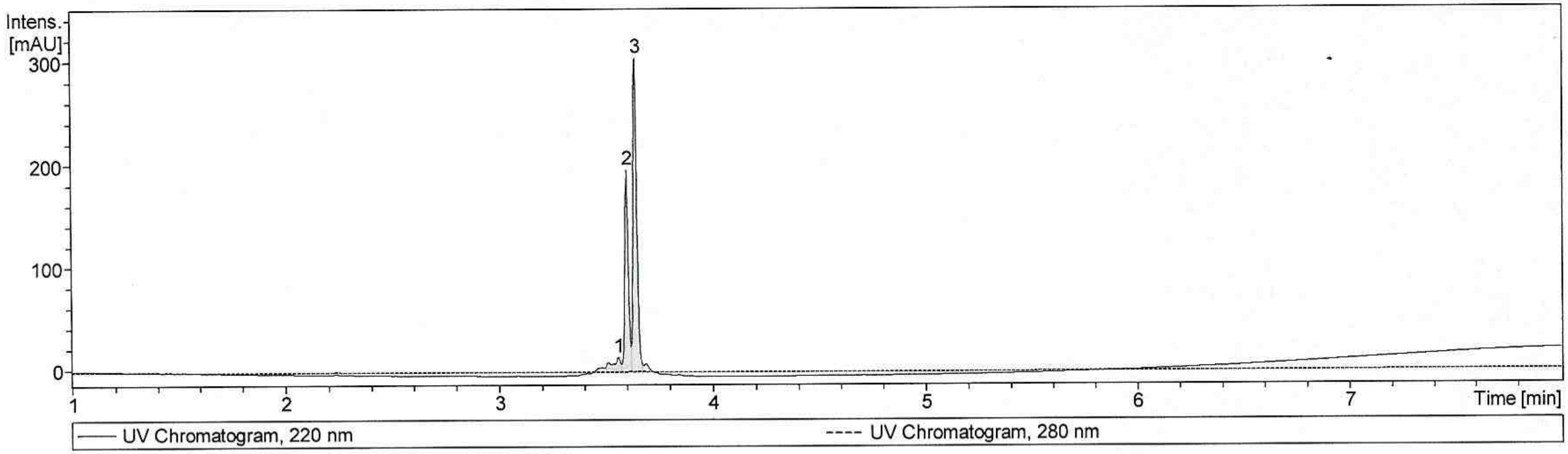

Target Mass	Meas. Mass	Expec. Mass	Delt. Mr [Da]	Intensity	Area	Area Fraction [%]
Cmpd 3; 3.64 min; Pep Mr: 2266.37	2266.37	2267.00	-0.63	302	372	59.4

#	RT [min]	Area	Area Frac. %
1	3.56	29.217	4.66
2	3.60	225.811	35.99
3	3.64	372.357	59.35

Peptide QC Report LB1503 33-41

Cmpd 2, 3.60 min

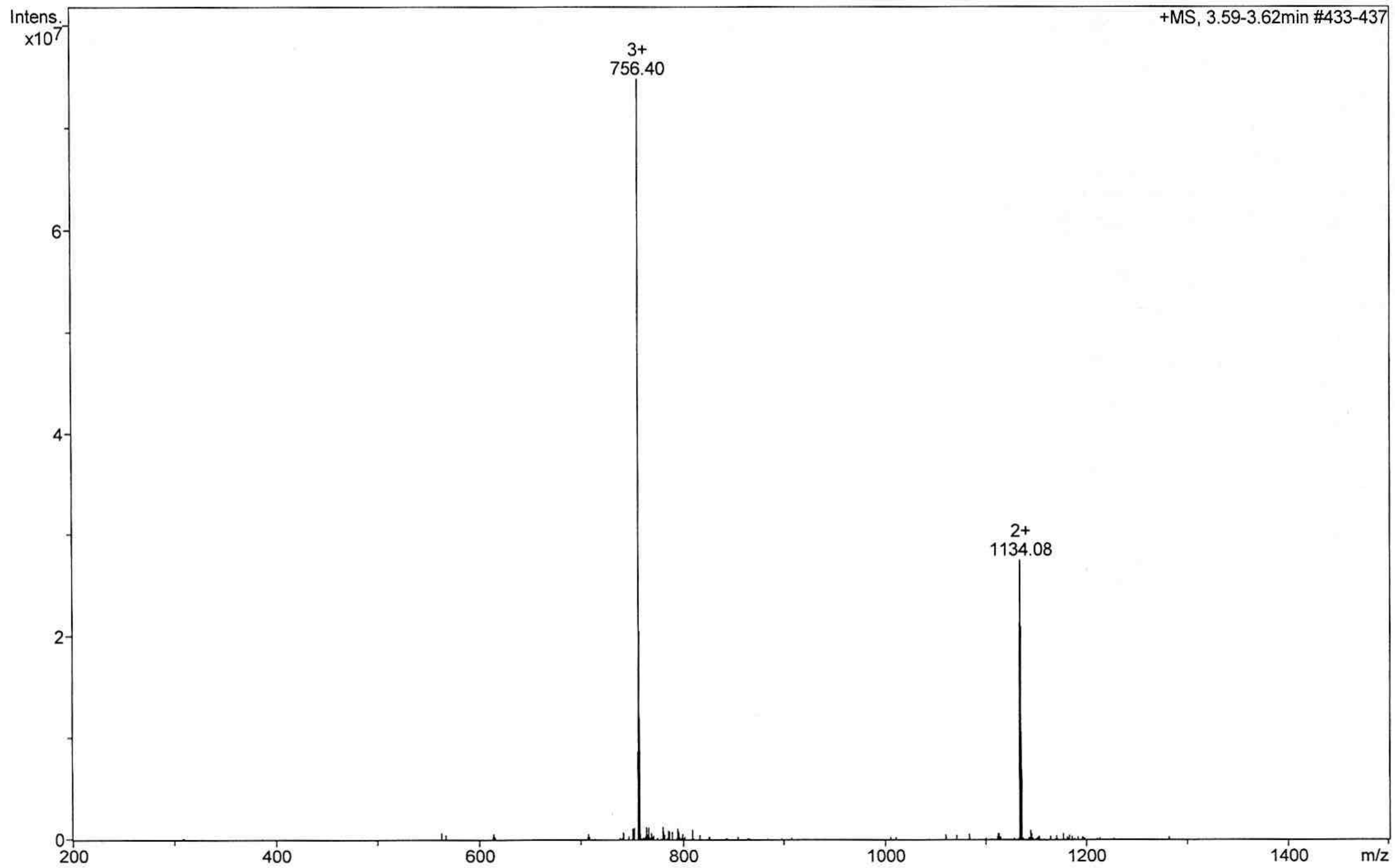

Peptide QC Report LB1503 33-41

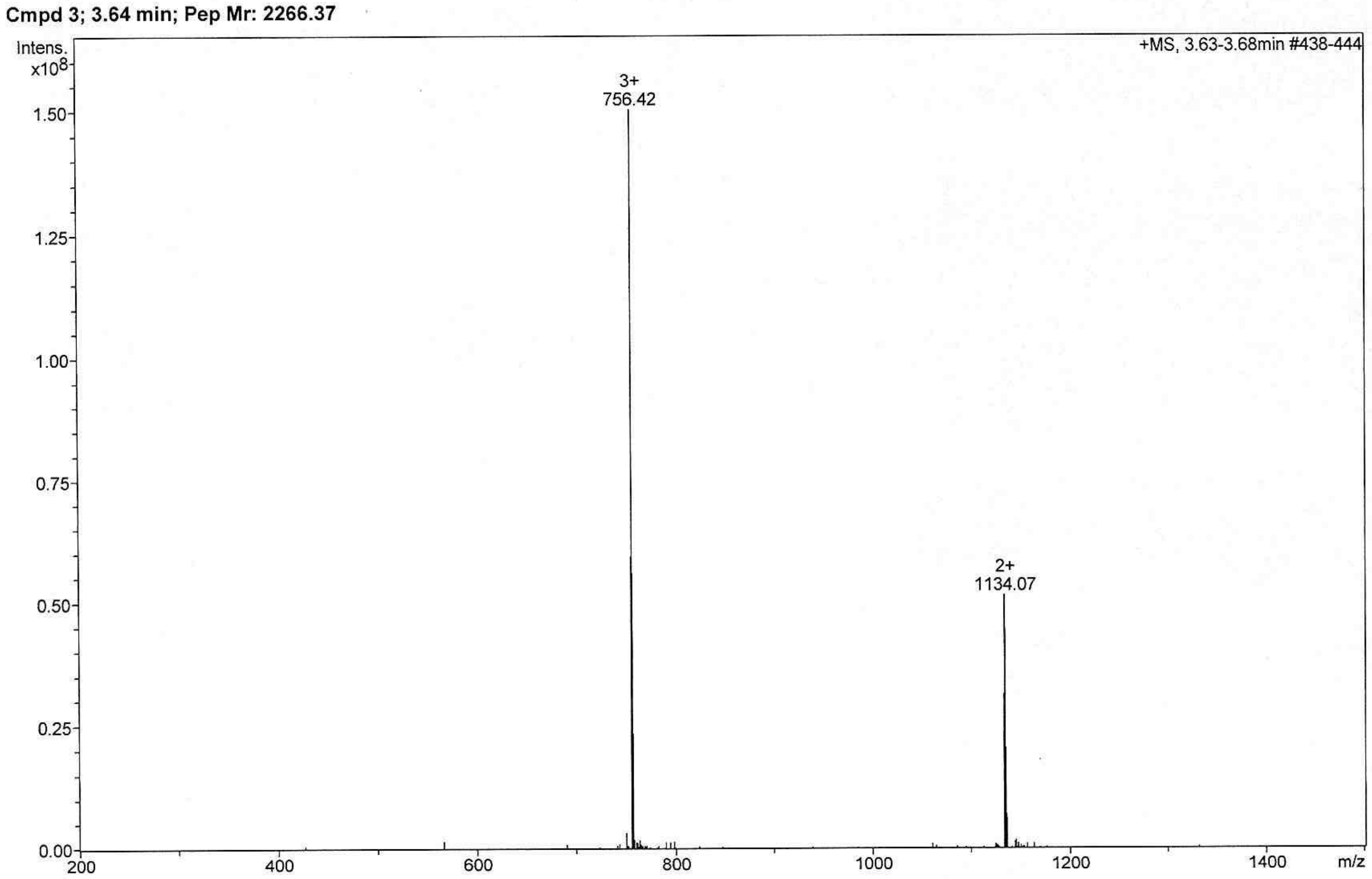

Supplement: Supplementary file 2 [file DataSheet2.PDF]
