# Supplementary material for: Screening of Hydrocarbon-Stapled Peptides for Inhibition of Calcium-Triggered Exocytosis
Source: Front Pharmacol. 2022 Jun 17;13:891041. doi: 10.3389/fphar.2022.891041 (PMC9258623; doi:10.3389/fphar.2022.891041)

new england peptide™

Certificate of Analysis

**Sequence:** [Cyc(5,9;12,16)]Ac-SKDA(S5)IRT(S5)VM(S5)DEQ(S5)EQL-amide	
**Peptide Name:**	**Date:** 8/7/2017

**Order#:** P611359	**Lot#:** LB1543	**Amount:** 5.1mg

Quality Control Specifications:

QC Test	QC Specifications	Results
Purity by HPLC	**≥90%** by percent area	**Pass**
Mass Identification by Mass Spectral Analysis	Calculated Mass within 0.1% of Molecular Weight: **2306**	**Pass**
Concentration/ Net Peptide	Amino Acid Analysis (AAA) determining original concentration/net peptide content.	**N/A**

Product: Research Grade Custom Peptide containing traces of Trifluoroacetate (TFA) salts.

Formulation:
Final concentration: N/A
Final form: Dry

Stability and Conditions: Refer to the Quality Control Detail Information on our website at www.newenglandpeptide.com/support/quality-control-information . As always, NEP has individual batch records stored electronically for each peptide that includes traceable lot numbers of raw materials used during synthesis. Should you require this information, email sales@newenglandpeptide.com with your peptide lot number.

Notes (if applicable):

Approval/Initials

For Science... From Science.

New England Peptide Inc., 65 Zub Lane, Gardner, MA 01440 ▪ **Phone** 888-343-5974 ▪ **Fax** 978-630-0021

www.NewEnglandPeptide.com

Peptide QC Report LB1543 36-47

Analysis Name	D:\Data\LB1543 36-47_143062_P1-D-9_01_71579.D
Sample Name	LB1543 36-47
Method	APRIL20171.2mLperMIN_NEPO AHIGH_71579.m
Instrument	amaZon SL

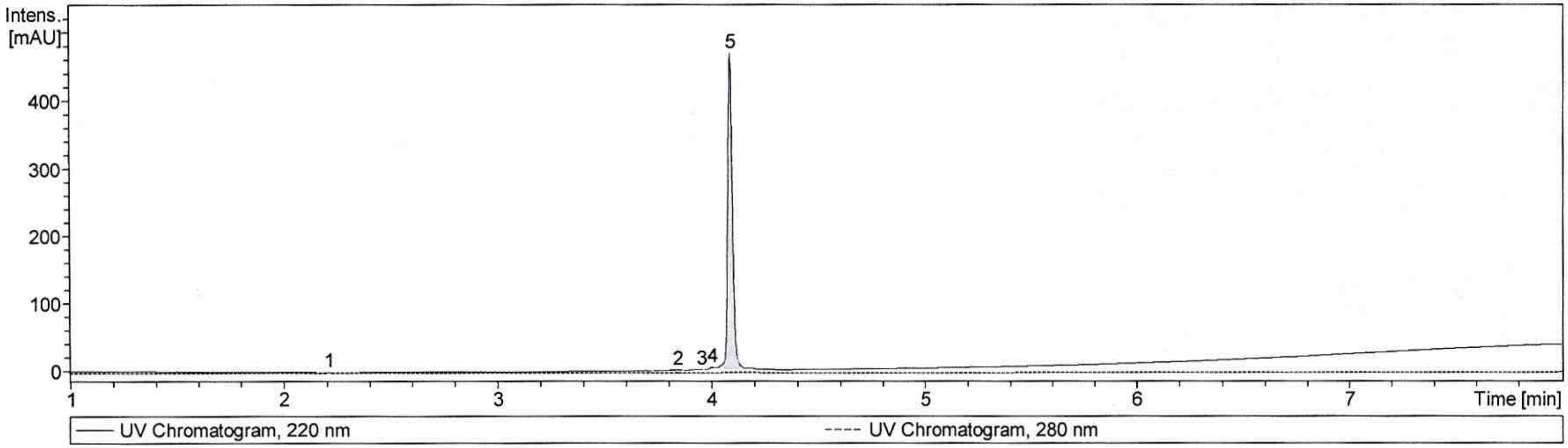

	Target Mass	Meas. Mass	Expec. Mass	Delt. Mr [Da]	Intensity	Area	Area Fraction [%]
Cmpd 5; 4.09 min; Pep Mr: 2305.12		2305.12	2306.00	-0.88	468	686	98.5

#	RT [min]	Area	Area Frac. %
1	2.21	1.9313	0.28
2	3.84	3.7429	0.54
3	3.95	2.1741	0.31
4	4.00	2.7394	0.39
5	4.09	685.6571	98.48

new england peptide

Peptide QC Report LB1543 36-47

Cmpd 5; 4.09 min; Pep Mr: 2305.12

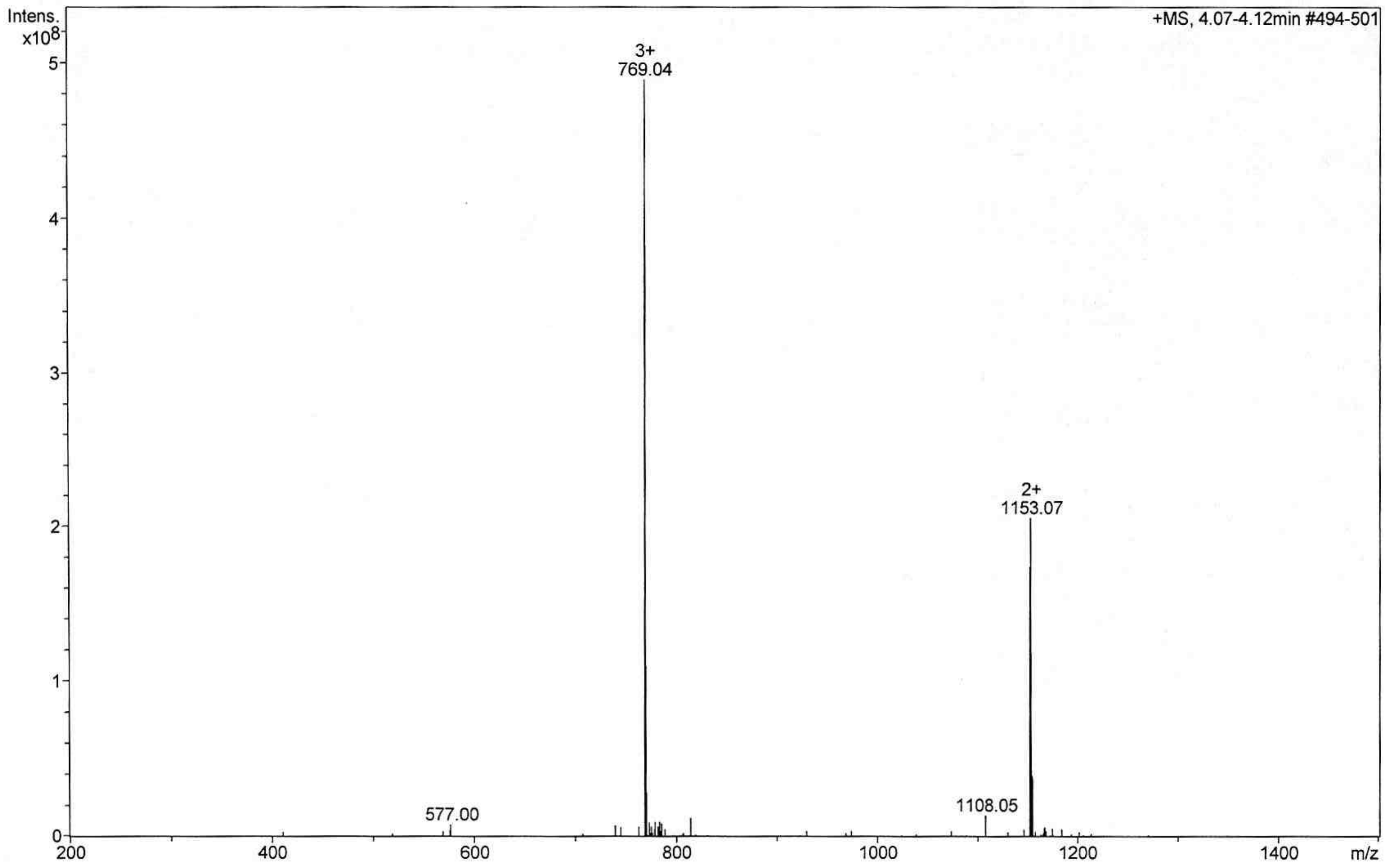

Supplement: Supplementary file 3 [file DataSheet4.PDF]
