# Supplementary material for: Screening of Hydrocarbon-Stapled Peptides for Inhibition of Calcium-Triggered Exocytosis
Source: Front Pharmacol. 2022 Jun 17;13:891041. doi: 10.3389/fphar.2022.891041 (PMC9258623; doi:10.3389/fphar.2022.891041)

new england peptide™

Certificate of Analysis

Sequence: [Cyc(9,16)]Ac-SKDAGIRT(R8)VMGDEQ(S5)EQL-amide

**Peptide Name:**	**Date:** 8/7/2017

**Order#:** P611359	**Lot#:** LB1501	**Amount:** 5.1mg

Quality Control Specifications:

QC Test	QC Specifications	Results
Purity by HPLC	≥**90%** by percent area	**Pass**
Mass Identification by Mass Spectral Analysis	Calculated Mass within 0.1% of Molecular Weight: **2211**	**Pass**
Concentration/ Net Peptide	Amino Acid Analysis (AAA) determining original concentration/net peptide content.	**N/A**

Product: Research Grade Custom Peptide containing traces of Trifluoroacetate (TFA) salts.

Formulation:
Final concentration: N/A
Final form: Dry

Stability and Conditions: Refer to the Quality Control Detail Information on our website at www.newenglandpeptide.com/support/quality-control-information . As always, NEP has individual batch records stored electronically for each peptide that includes traceable lot numbers of raw materials used during synthesis. Should you require this information, email sales@newenglandpeptide.com with your peptide lot number.

Notes (if applicable):

Approval/Initials

Peptide QC Report LB1501 116-131

Analysis Name	D:\Data\LB1501 116-131_143056_P1-D-4_01_71576.D
Sample Name	LB1501 116-131
Method	APRIL20171.2mLperMIN_NEPOAHIGH_71576.m
Instrument	amaZon SL

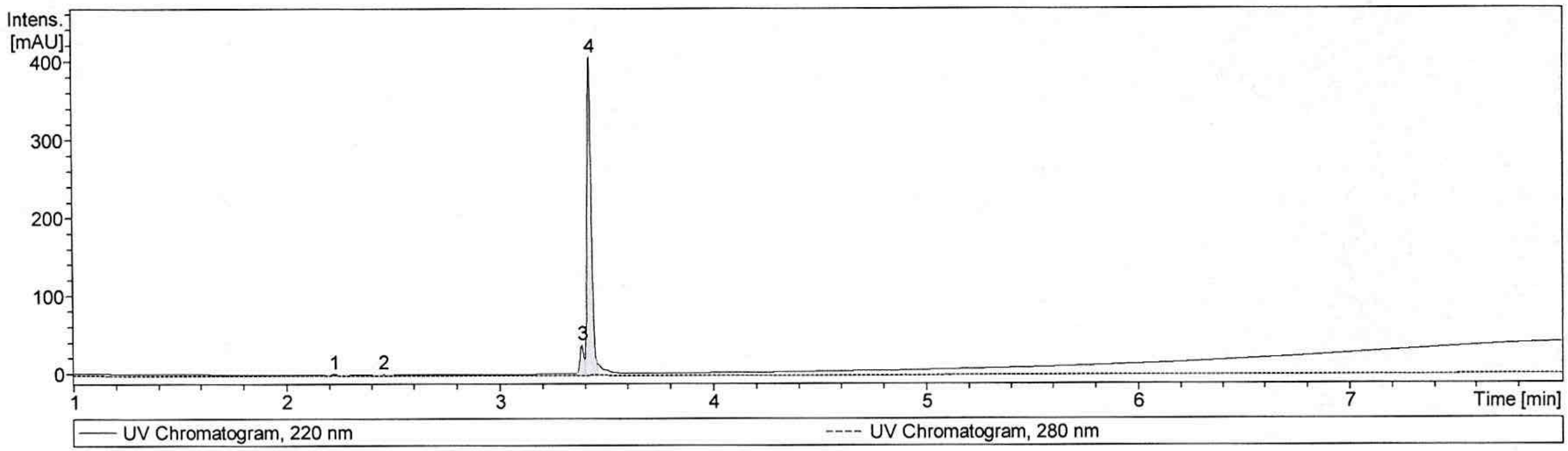

Target Mass	Meas. Mass	Expec. Mass	Delt. Mr [Da]	Intensity	Area	Area Fraction [%]
Cmpd 4; 3.42 min; Pep Mr: 2210.14	2210.14	2211.00	-0.86	404	570	92.4

#	RT [min]	Area	Area Frac. %
1	2.23	1.5397	0.25
2	2.46	0.9531	0.15
3	3.39	44.5118	7.22
4	3.42	569.7297	92.38

Peptide QC Report LB1501 116-131

Cmpd 4; 3.42 min; Pep Mr: 2210.14

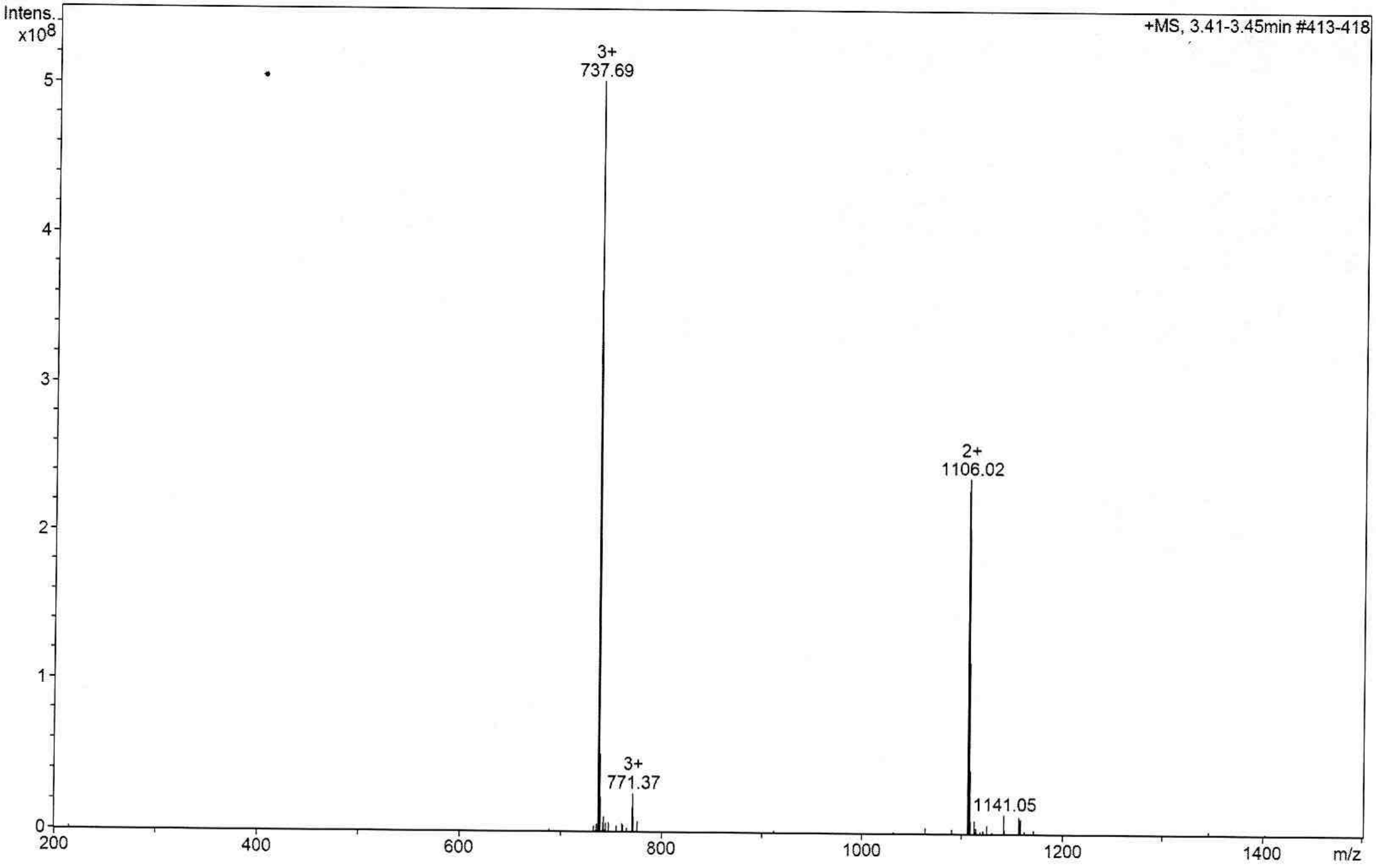

Supplement: Supplementary file 4 [file DataSheet6.PDF]
