# Supplementary material for: Screening of Hydrocarbon-Stapled Peptides for Inhibition of Calcium-Triggered Exocytosis
Source: Front Pharmacol. 2022 Jun 17;13:891041. doi: 10.3389/fphar.2022.891041 (PMC9258623; doi:10.3389/fphar.2022.891041)

new england peptide™

# Certificate of Analysis

| **Sequence:** [Cyc(3,7;10,14)]Ac-EE(S5)KDA(S5)IR(S5)LVM(S5)DEQC-amide |
|---|

| **Peptide Name:** | **Date:** 8/7/2017 |
|---|---|

| **Order#:** P611359 | **Lot#:** LB1544 | **Amount:** 5.4mg |
|---|---|---|

**Quality Control Specifications:**

| QC Test | QC Specifications | Results |
|---|---|---|
| Purity by HPLC | **≥90%** by percent area | **Pass** |
| Mass Identification by Mass Spectral Analysis | Calculated Mass within 0.1% of Molecular Weight: **2222** | **Pass** |
| Concentration/ Net Peptide | Amino Acid Analysis (AAA) determining original concentration/net peptide content. | **N/A** |

**Product:** Research Grade Custom Peptide containing traces of Trifluoroacetate (TFA) salts.

**Formulation:**
Final concentration: N/A
Final form: Dry

**Stability and Conditions:** Refer to the Quality Control Detail Information on our website at www.newenglandpeptide.com/support/quality-control-information . As always, NEP has individual batch records stored electronically for each peptide that includes traceable lot numbers of raw materials used during synthesis. Should you require this information, email sales@newenglandpeptide.com with your peptide lot number.

**Notes (if applicable):**

JM

**Approval/Initials**

# Peptide QC Report LB1544 25-43

| | |
|---|---|
| Analysis Name | D:\Data\LB1544 25-43_143070_P1-E-5_01_71582.D |
| Sample Name | LB1544 25-43 |
| Method | APRIL20171.2mLperMIN_NEPO AHIGH_71582.m |
| Instrument | amaZon SL |

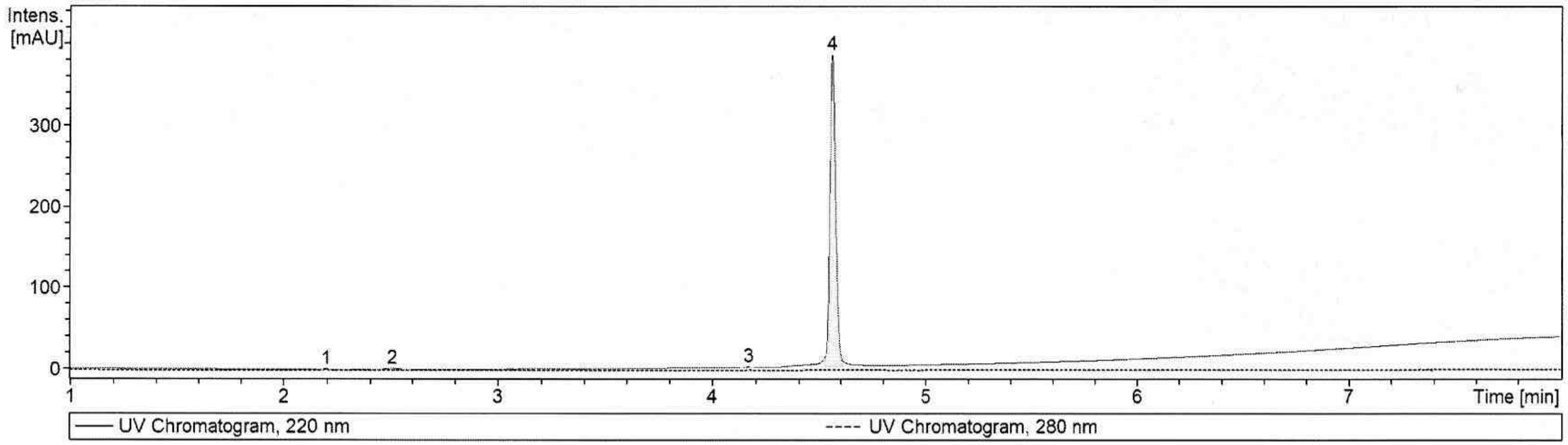


| Target Mass | Meas. Mass | Expec. Mass | Delt. Mr [Da] | Intensity | Area | Area Fraction [%] |
|---|---|---|---|---|---|---|
| Cmpd 4; 4.56 min; Pep Mr: 2221.04 | 2221.04 | 2222.00 | -0.96 | 386 | 713 | 98.8 |

| # | RT [min] | Area | Area Frac. % |
|---|---|---|---|
| 1 | 2.20 | 1.7029 | 0.24 |
| 2 | 2.50 | 5.3905 | 0.75 |
| 3 | 4.17 | 1.2394 | 0.17 |
| 4 | 4.56 | 712.5846 | 98.84 |

# Peptide QC Report LB1544 25-43

Cmpd 4; 4.56 min; Pep Mr: 2221.04

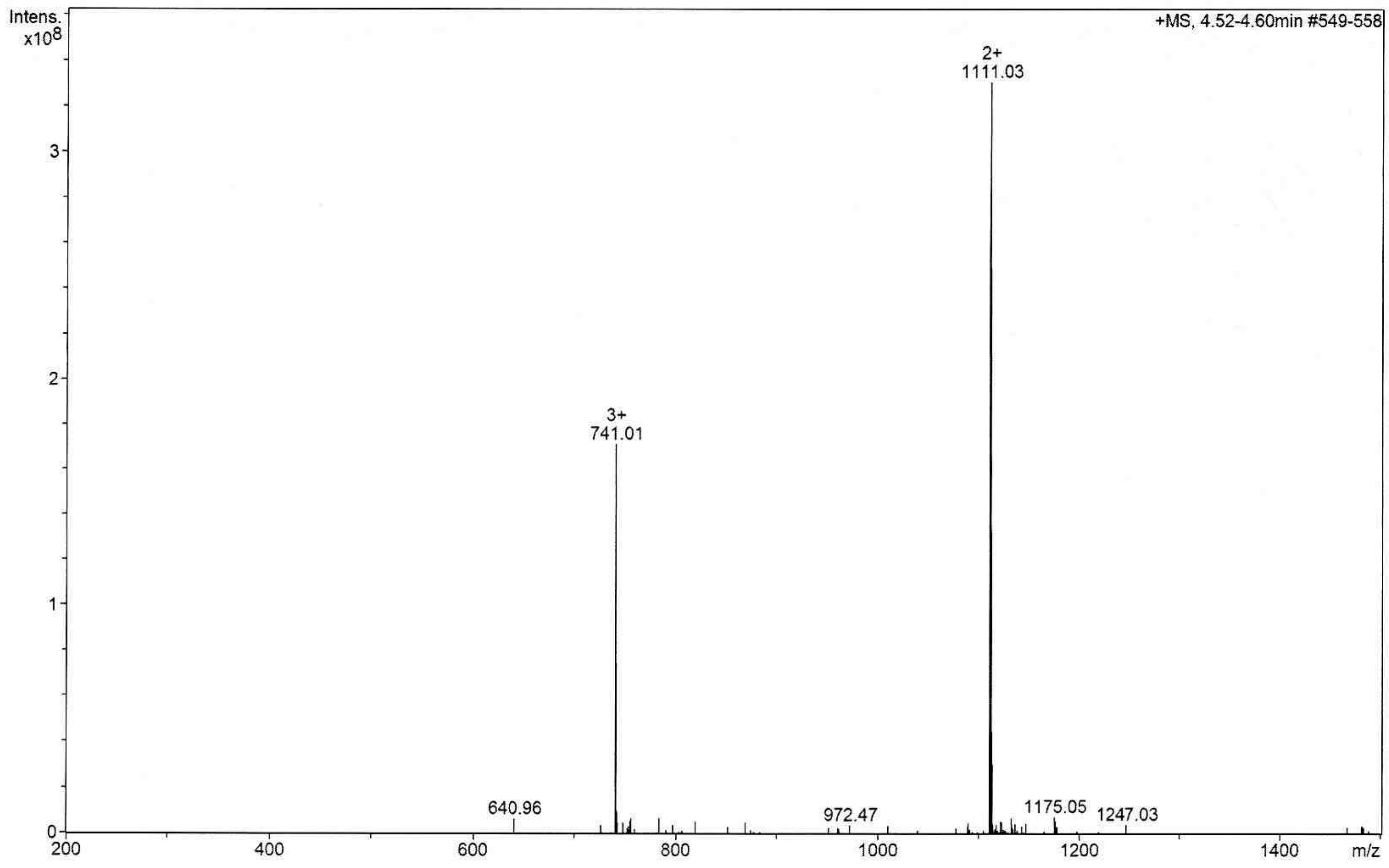

Supplement: Supplementary file 5 [file DataSheet9.PDF]
