# Supplementary material for: Screening of Hydrocarbon-Stapled Peptides for Inhibition of Calcium-Triggered Exocytosis
Source: Front Pharmacol. 2022 Jun 17;13:891041. doi: 10.3389/fphar.2022.891041 (PMC9258623; doi:10.3389/fphar.2022.891041)

new england peptide™

Certificate of Analysis

**Sequence:** [Cyc(5,9;11,15)]Ac-SKDA(S5)IRT(S5)V(S5)LDE(S5)GEQL-amide

**Peptide Name:**	**Date:** 8/9/2017

**Order#:** P611359	**Lot#:** LB1541	**Amount:** 5.2mg

Quality Control Specifications:

QC Test	QC Specifications	Results
Purity by HPLC	≥**90%** by percent area	**Pass**
Mass Identification by Mass Spectral Analysis	Calculated Mass within 0.1% of Molecular Weight: **2217**	**Pass**
Concentration/ Net Peptide	Amino Acid Analysis (AAA) determining original concentration/net peptide content.	**N/A**

Product: Research Grade Custom Peptide containing traces of Trifluoroacetate (TFA) salts.

Formulation:
Final concentration: N/A
Final form: Dry

Stability and Conditions: Refer to the Quality Control Detail Information on our website at www.newenglandpeptide.com/support/quality-control-information . As always, NEP has individual batch records stored electronically for each peptide that includes traceable lot numbers of raw materials used during synthesis. Should you require this information, email sales@newenglandpeptide.com with your peptide lot number.

Notes (if applicable):

BS
Approval/Initials

For Science... From Science.

New England Peptide Inc., 65 Zub Lane, Gardner, MA 01440 ▪ **Phone** 888-343-5974 ▪ **Fax** 978-630-0021

www.NewEnglandPeptide.com

Peptide QC Report LB1541 32-36

Analysis Name	D:\Data\LB1541 32-36_143575_P1-F-8_01_71819.D
Sample Name	LB1541 32-36
Method	APRIL20171.2mLperMIN_NEPO AHIGH_71819.m
Instrument	amaZon SL

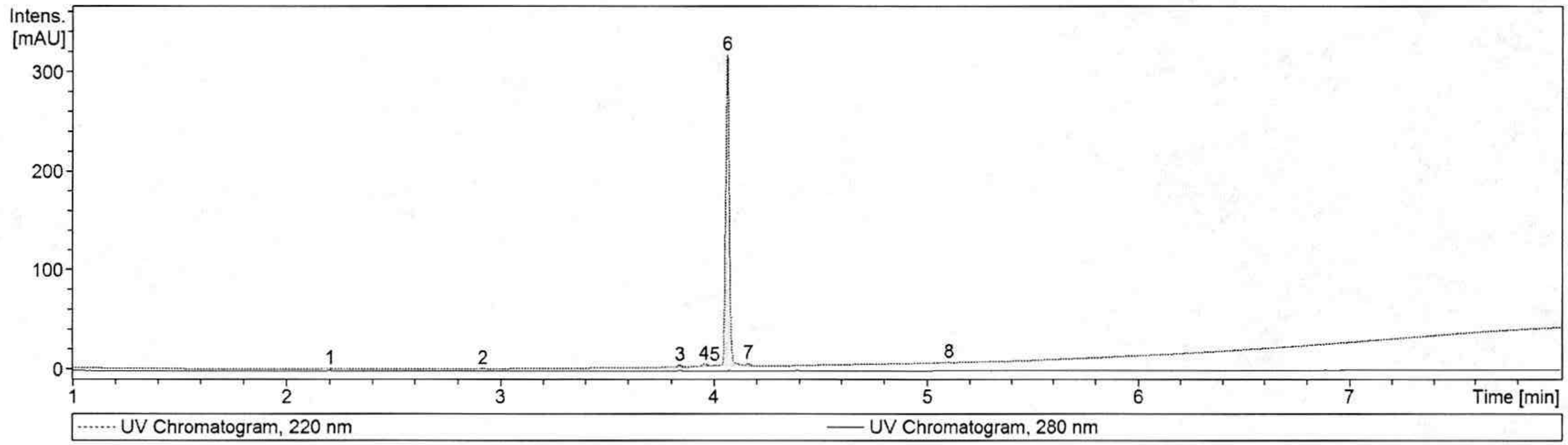

Target Mass	Meas. Mass	Expec. Mass	Delt. Mr [Da]	Intensity	Area	Area Fraction [%]
Cmpd 6; 4.06 min; Pep Mr: 2216.09	2216.09	2217.00	-0.91	316	369	96.3

#	RT [min]	Area	Area Frac. %
1	2.20	1.9386	0.51
2	2.92	0.5557	0.15
3	3.84	3.2859	0.86
4	3.96	4.3244	1.13
5	4.00	0.6521	0.17

#	RT [min]	Area	Area Frac. %
6	4.06	368.6080	96.25
7	4.16	2.3079	0.60
8	5.10	1.2776	0.33

Cmpd 6; 4.06 min; Pep Mr: 2216.09

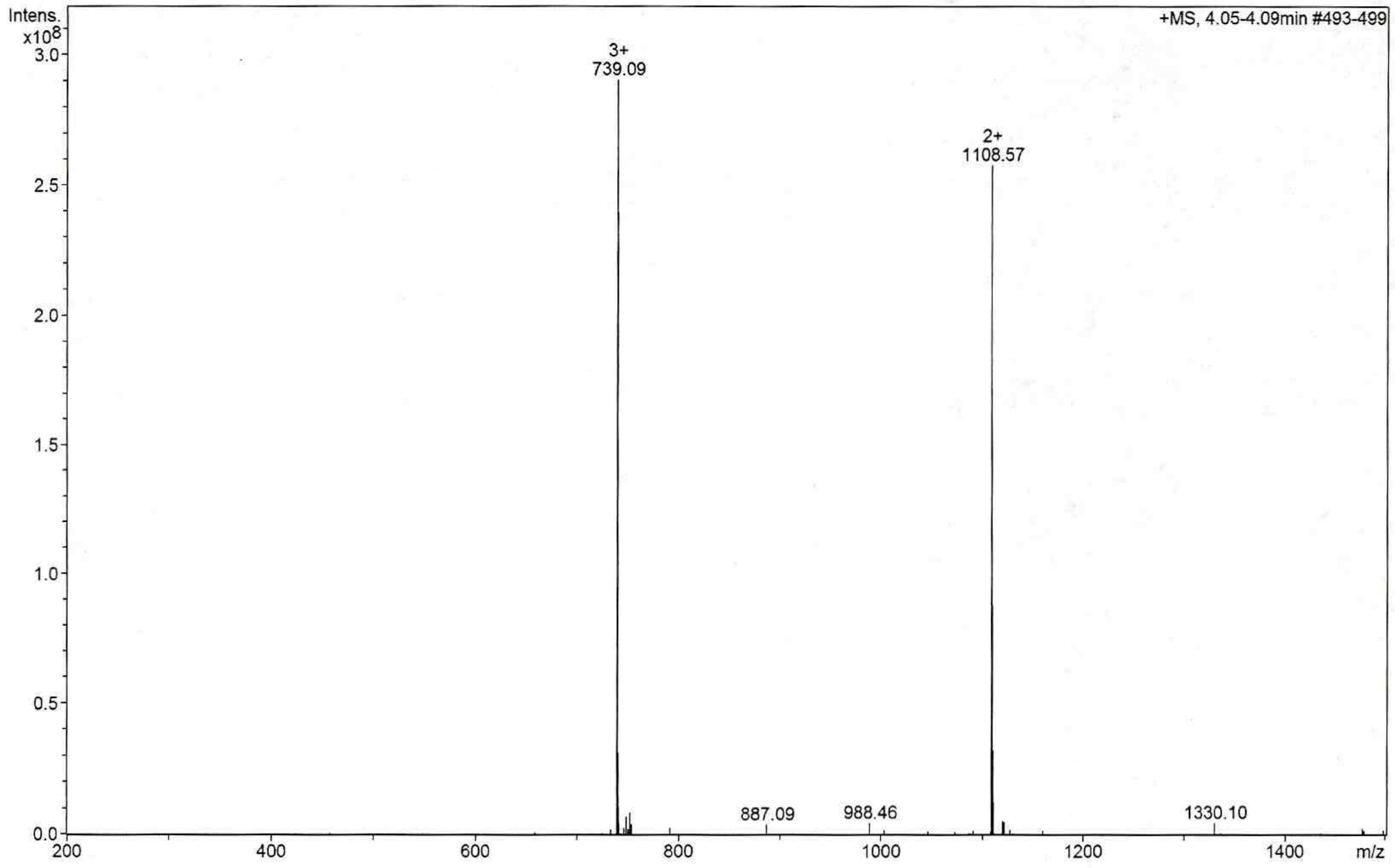

Supplement: Supplementary file 7 [file DataSheet11.PDF]
