# Supplementary material for: Screening of Hydrocarbon-Stapled Peptides for Inhibition of Calcium-Triggered Exocytosis
Source: Front Pharmacol. 2022 Jun 17;13:891041. doi: 10.3389/fphar.2022.891041 (PMC9258623; doi:10.3389/fphar.2022.891041)

new england peptide™

# Certificate of Analysis

| | | |
|---|---|---|
| **Sequence:** [Cyc(5,12)]Ac-SKDA(R8)IRGLVM(S5)DEQC-amide | | |
| **Peptide Name:** | | **Date:** 8/8/2017 |
| **Order#:** P611359 | **Lot#:** LB1504 | **Amount:** 5.0mg |

**Quality Control Specifications:**

| QC Test | QC Specifications | Results |
|---|---|---|
| Purity by HPLC | ≥**90%** by percent area | **Pass** |
| Mass Identification by Mass Spectral Analysis | Calculated Mass within 0.1% of Molecular Weight: **1899** | **Pass** |
| Concentration/ Net Peptide | Amino Acid Analysis (AAA) determining original concentration/net peptide content. | **N/A** |

**Product:** Research Grade Custom Peptide containing traces of Trifluoroacetate (TFA) salts.

**Formulation:**
Final concentration: N/A
Final form: Dry

**Stability and Conditions:** Refer to the Quality Control Detail Information on our website at www.newenglandpeptide.com/support/quality-control-information . As always, NEP has individual batch records stored electronically for each peptide that includes traceable lot numbers of raw materials used during synthesis. Should you require this information, email sales@newenglandpeptide.com with your peptide lot number.

**Notes (if applicable):**

JM

**Approval/Initials**

*For Science... From Science.*

New England Peptide Inc., 65 Zub Lane, Gardner, MA 01440 ▪ **Phone** 888-343-5974 ▪ **Fax** 978-630-0021

**www.NewEnglandPeptide.com**

new england peptide

# Peptide QC Report LB1504 58-76

| | |
|---|---|
| Analysis Name | D:\Data\LB1504 58-76_143099_P1-F-6_01_71591.D |
| Sample Name | LB1504 58-76 |
| Method | APRIL20171.2mLperMIN_NEPO AHIGH_71591.m |
| Instrument | amaZon SL |

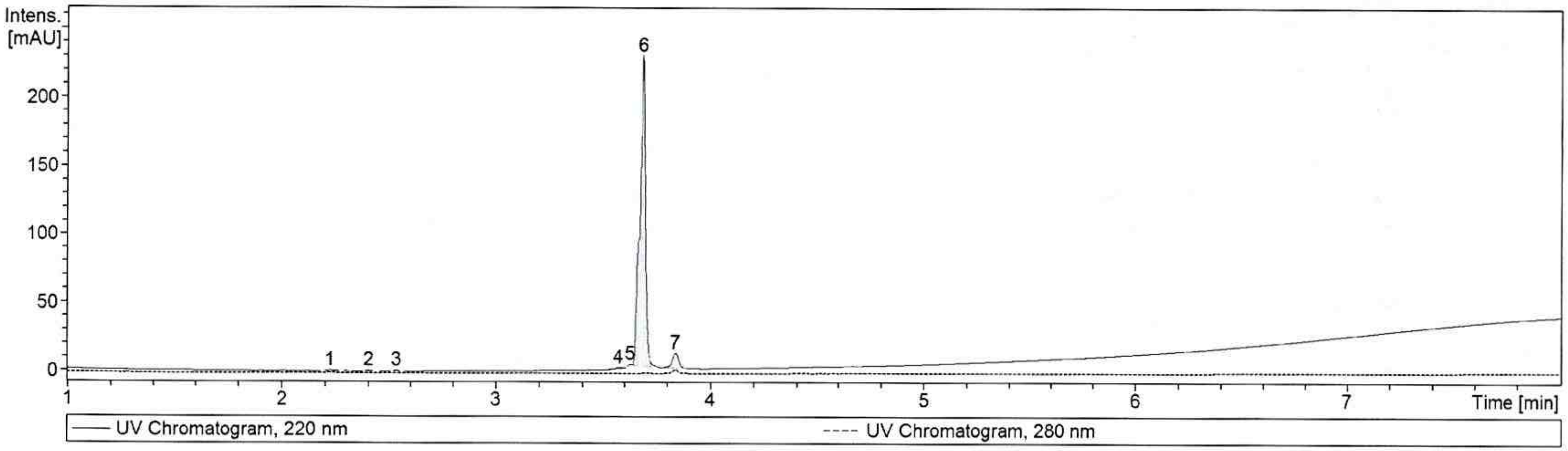


| Target Mass | Meas. Mass | Expec. Mass | Delt. Mr [Da] | Intensity | Area | Area Fraction [%] |
|---|---|---|---|---|---|---|
| Cmpd 6; 3.68 min; Pep Mr: 1898.92 | 1898.92 | 1899.00 | -0.08 | 229 | 402 | 93.2 |

| # | RT [min] | Area | Area Frac. % |
|---|---|---|---|
| 1 | 2.22 | 1.6955 | 0.39 |
| 2 | 2.40 | 0.8997 | 0.21 |
| 3 | 2.53 | 0.9458 | 0.22 |
| 4 | 3.57 | 2.0056 | 0.46 |
| 5 | 3.63 | 1.4264 | 0.33 |

| # | RT [min] | Area | Area Frac. % |
|---|---|---|---|
| 6 | 3.68 | 402.3632 | 93.21 |
| 7 | 3.84 | 22.3181 | 5.17 |

# Peptide QC Report    LB1504 58-76

Cmpd 6; 3.68 min; Pep Mr: 1898.92

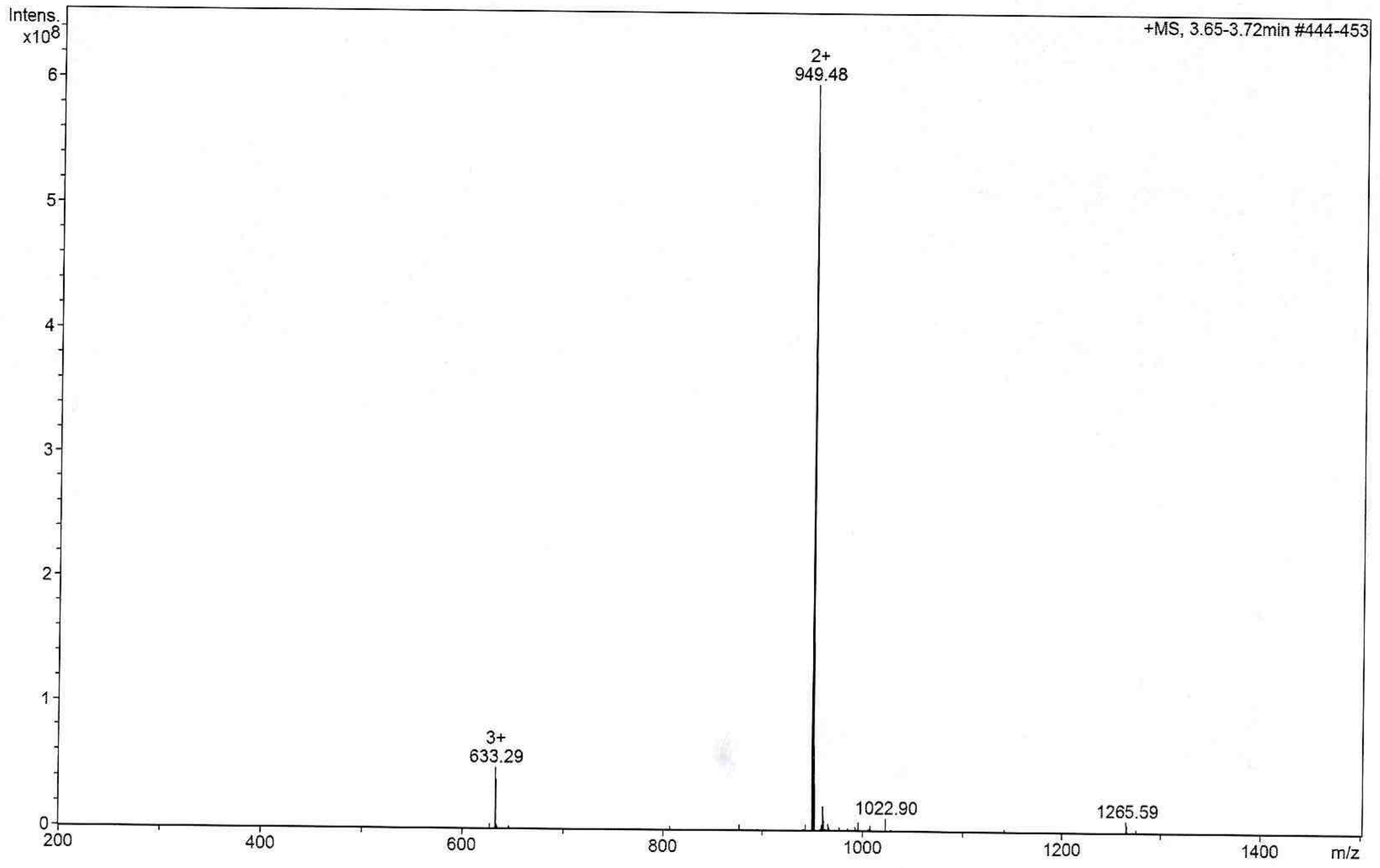

Supplement: Supplementary file 8 [file DataSheet3.PDF]
