# Supplementary material for: Screening of Hydrocarbon-Stapled Peptides for Inhibition of Calcium-Triggered Exocytosis
Source: Front Pharmacol. 2022 Jun 17;13:891041. doi: 10.3389/fphar.2022.891041 (PMC9258623; doi:10.3389/fphar.2022.891041)

new england peptide™

Certificate of Analysis

**Sequence:** [Cyc(4,8;15,19)]Ac-SKD(S5)GIR(S5)LVMLDE(S5)GEQ(S5)DR-amide

**Peptide Name:**	**Date:** 8/7/2017

**Order#:** P611359	**Lot#:** LB1505	**Amount:** 5.2mg

Quality Control Specifications:

QC Test	QC Specifications	Results
Purity by HPLC	≥**90%** by percent area	**Pass**
Mass Identification by Mass Spectral Analysis	Calculated Mass within 0.1% of Molecular Weight: **2504**	**Pass**
Concentration/ Net Peptide	Amino Acid Analysis (AAA) determining original concentration/net peptide content.	**N/A**

Product: Research Grade Custom Peptide containing traces of Trifluoroacetate (TFA) salts.

Formulation:
Final concentration: N/A
Final form: Dry

Stability and Conditions: Refer to the Quality Control Detail Information on our website at www.newenglandpeptide.com/support/quality-control-information . As always, NEP has individual batch records stored electronically for each peptide that includes traceable lot numbers of raw materials used during synthesis. Should you require this information, email sales@newenglandpeptide.com with your peptide lot number.

Notes (if applicable):

JM

Approval/Initials

new england peptide

Peptide QC Report LB1505 105-115

Analysis Name	D:\Data\LB1505 105-115_143031_P1-A-9_01_71557.d
Sample Name	LB1505 105-115
Method	APRIL20171.2mLperMIN_NEPO AHIGH_71557.m
Instrument	amaZon SL

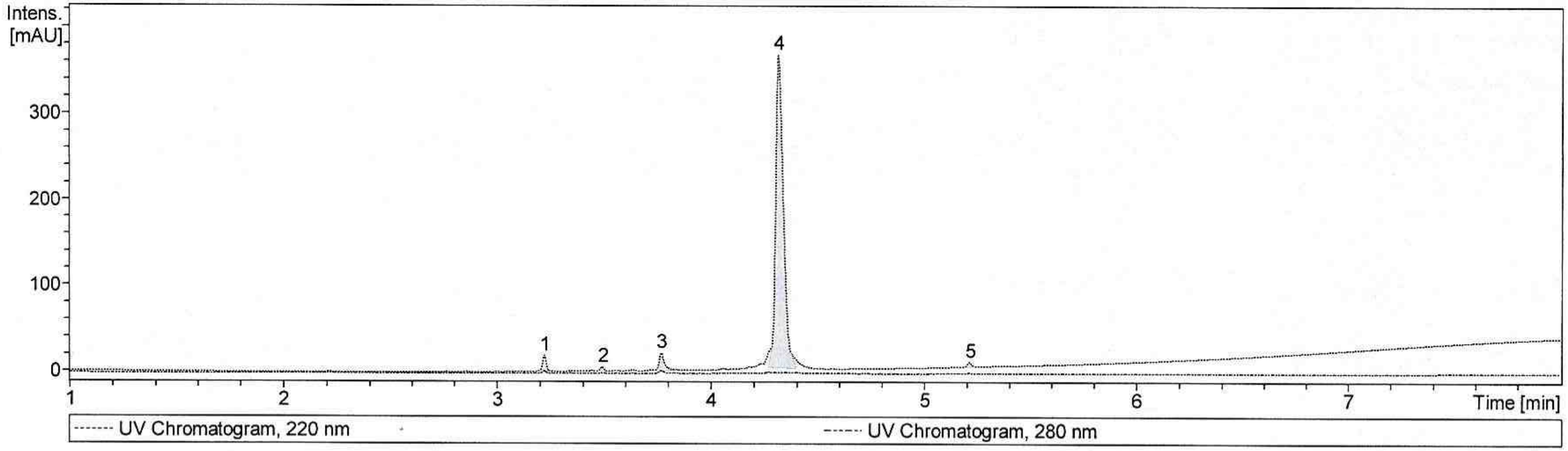

Target Mass	Meas. Mass	Expec. Mass	Delt. Mr [Da]	Intensity	Area	Area Fraction [%]
Cmpd 4; 4.31 min; Pep Mr: 2503.17	2503.17	2504.00	-0.83	367	899	92.7

#	RT [min]	Area	Area Frac. %
1	3.22	22.0159	2.27
2	3.49	6.3041	0.65
3	3.77	35.2296	3.63
4	4.31	899.0390	92.74
5	5.21	6.8335	0.70

Peptide QC Report LB1505 105-115

Cmpd 4; 4.31 min; Pep Mr: 2503.17

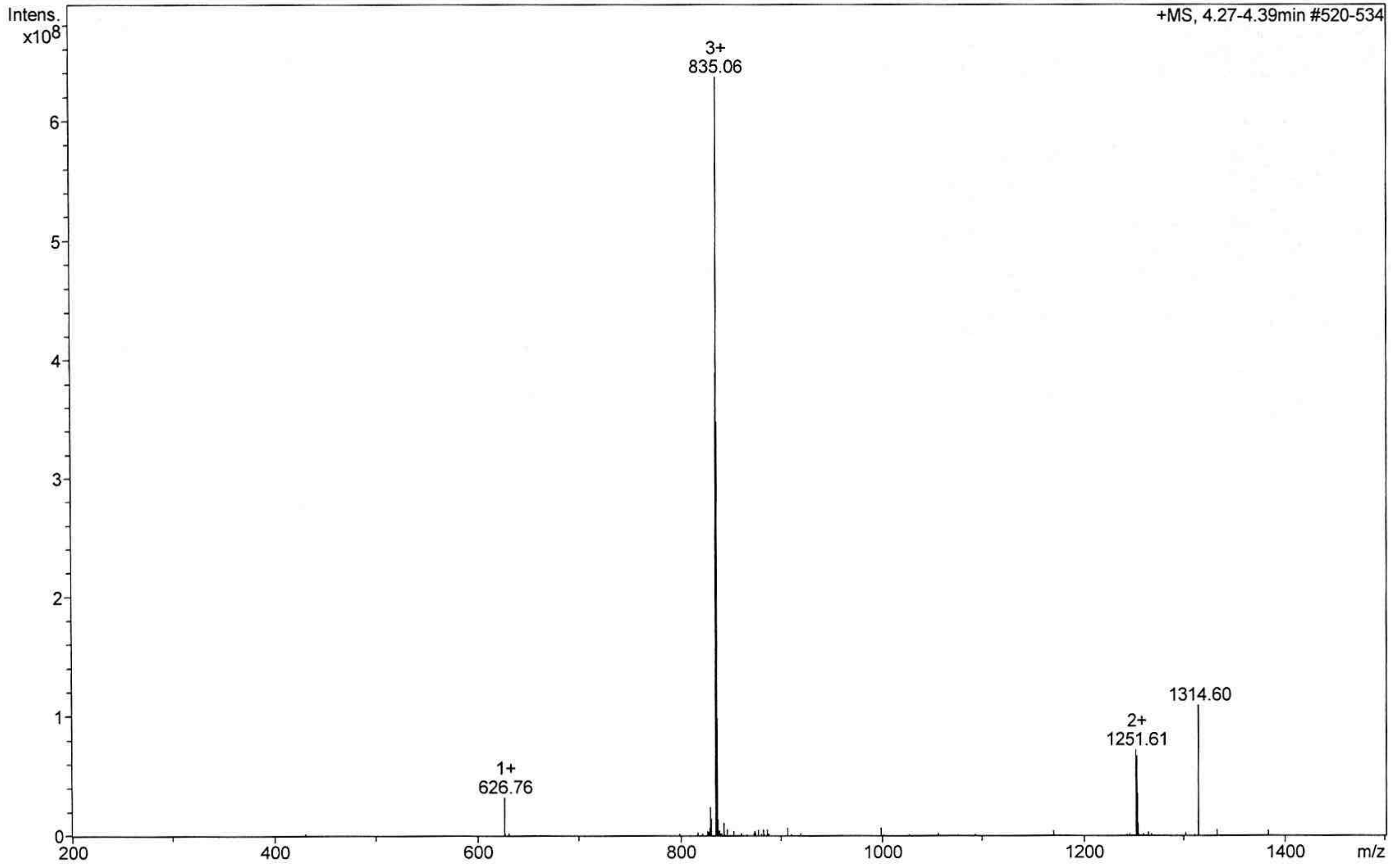

Supplement: Supplementary file 9 [file DataSheet1.PDF]
