# Supplementary material for: Screening of Hydrocarbon-Stapled Peptides for Inhibition of Calcium-Triggered Exocytosis
Source: Front Pharmacol. 2022 Jun 17;13:891041. doi: 10.3389/fphar.2022.891041 (PMC9258623; doi:10.3389/fphar.2022.891041)

new england peptide™

Certificate of Analysis

**Sequence:** [Cyc(8,15)]Ac-SKDAGIR(R8)LVMGDE(S5)GEQL-amide

**Peptide Name:**	**Date:** 8/7/2017

**Order#:** P611359	**Lot#:** LB1502	**Amount:** 5.1mg

Quality Control Specifications:

QC Test	QC Specifications	Results
Purity by HPLC	$\geq$**90%** by percent area	**Pass**
Mass Identification by Mass Spectral Analysis	Calculated Mass within 0.1% of Molecular Weight: **2152**	**Pass**
Concentration/ Net Peptide	Amino Acid Analysis (AAA) determining original concentration/net peptide content.	**N/A**

Product: Research Grade Custom Peptide containing traces of Trifluoroacetate (TFA) salts.

Formulation:
Final concentration: N/A
Final form: Dry

Stability and Conditions: Refer to the Quality Control Detail Information on our website at www.newenglandpeptide.com/support/quality-control-information . As always, NEP has individual batch records stored electronically for each peptide that includes traceable lot numbers of raw materials used during synthesis. Should you require this information, email sales@newenglandpeptide.com with your peptide lot number.

Notes (if applicable):

JM

Approval/Initials

Peptide QC Report   LB1502 60-69

Analysis Name	D:\Data\LB1502 60-69_143093_P1-C-7_01_76830.D
Sample Name	LB1502 60-69
Method	APRIL20171.2mLperMIN_NEPOAHIGH_76830.m
Instrument	amaZon SL

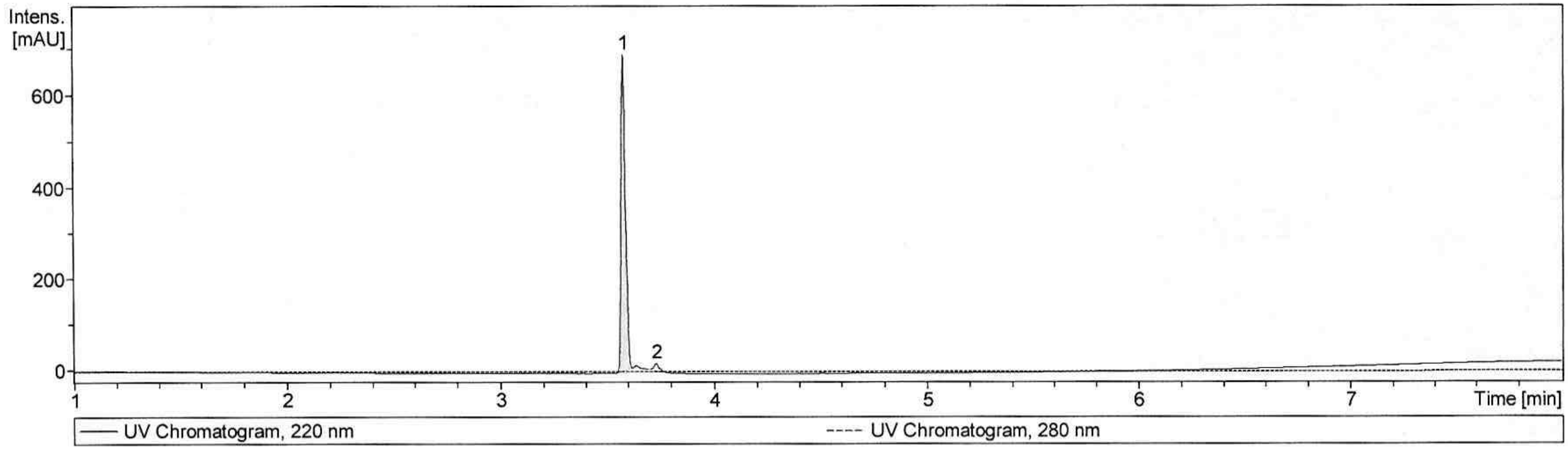

Target Mass	Meas. Mass	Expec. Mass	Delt. Mr [Da]	Intensity	Area	Area Fraction [%]
Cmpd 1; 3.58 min; Pep Mr: 2152.17	2152.17	2152.00	0.17	686	957	97.2

#	RT [min]	Area	Area Frac. %
1	3.58	957.307	97.21
2	3.73	27.452	2.79

Peptide QC Report LB1502 60-69

Cmpd 1; 3.58 min; Pep Mr: 2152.17

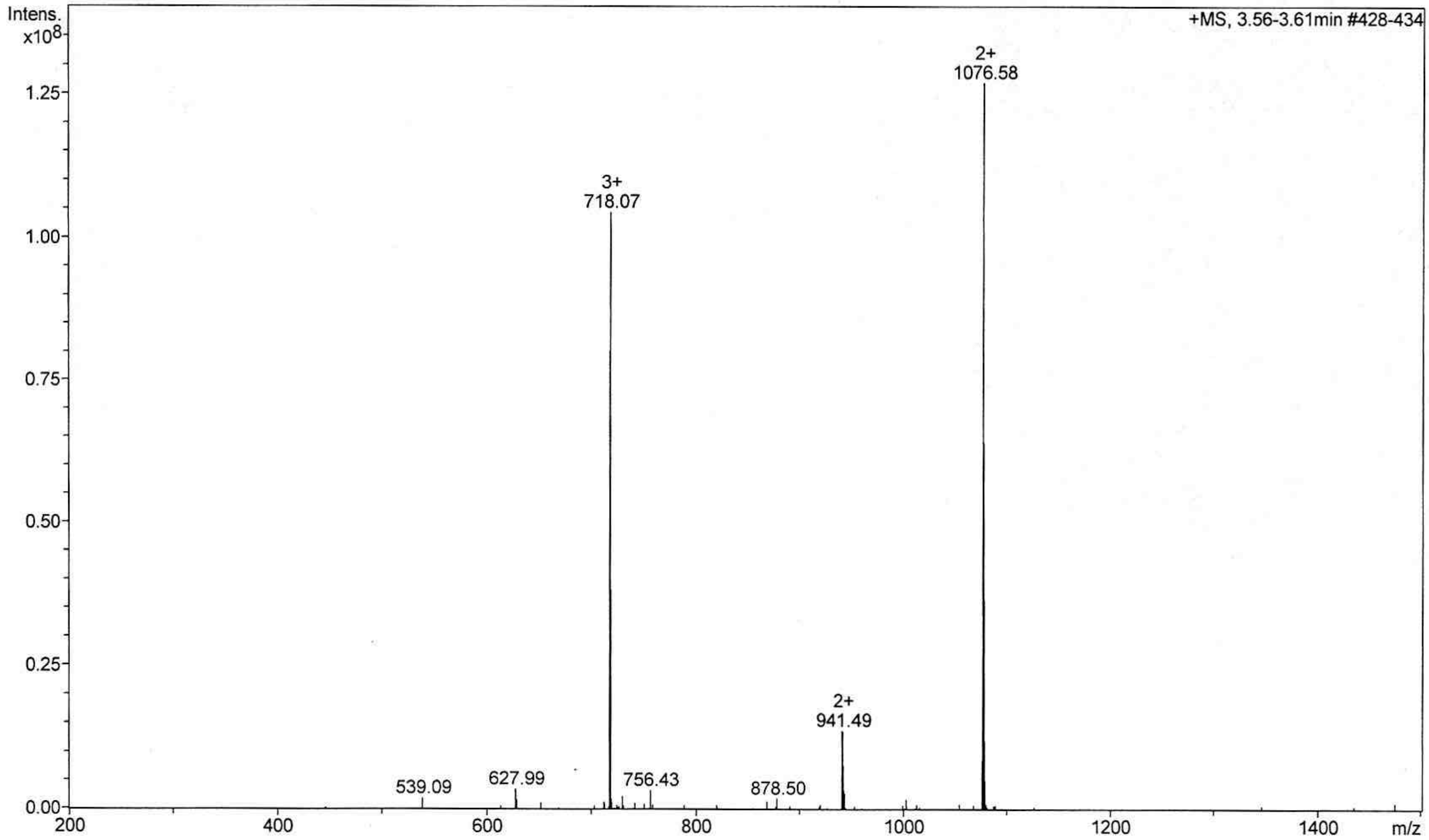

Supplement: Supplementary file 10 [file DataSheet5.PDF]
