# Supplementary material for: Screening of Hydrocarbon-Stapled Peptides for Inhibition of Calcium-Triggered Exocytosis
Source: Front Pharmacol. 2022 Jun 17;13:891041. doi: 10.3389/fphar.2022.891041 (PMC9258623; doi:10.3389/fphar.2022.891041)

nep
new england peptide™

Certificate of Analysis

**Sequence:** [Cyc(4,11)]Ac-SKD(R8)GIRGLV(S5)LDEQC-amide		
**Peptide Name:**		**Date:** 8/8/2017
**Order#:** P611359	**Lot#:** LB1429	**Amount:** 5.1mg

Quality Control Specifications:

QC Test	QC Specifications	Results
Purity by HPLC	≥**90%** by percent area	**Pass**
Mass Identification by Mass Spectral Analysis	Calculated Mass within 0.1% of Molecular Weight: **1867**	**Pass**
Concentration/ Net Peptide	Amino Acid Analysis (AAA) determining original concentration/net peptide content.	**N/A**

Product: Research Grade Custom Peptide containing traces of Trifluoroacetate (TFA) salts.

Formulation:
Final concentration: N/A
Final form: Dry

Stability and Conditions: Refer to the Quality Control Detail Information on our website at www.newenglandpeptide.com/support/quality-control-information . As always, NEP has individual batch records stored electronically for each peptide that includes traceable lot numbers of raw materials used during synthesis. Should you require this information, email sales@newenglandpeptide.com with your peptide lot number.

Notes (if applicable): Both isomers will be counted towards final purity.

Approval/Initials

For Science... From Science.

New England Peptide Inc., 65 Zub Lane, Gardner, MA 01440 ▪ **Phone** 888-343-5974 ▪ **Fax** 978-630-0021

www.NewEnglandPeptide.com

Peptide QC Report LB1429 16-34

Analysis Name	D:\Data\LB1429 16-34_142897_P1-D-6_01_76716.D
Sample Name	LB1429 16-34
Method	APRIL20171.2mLperMIN_NEPOAHIGH_76716.m
Instrument	amaZon SL

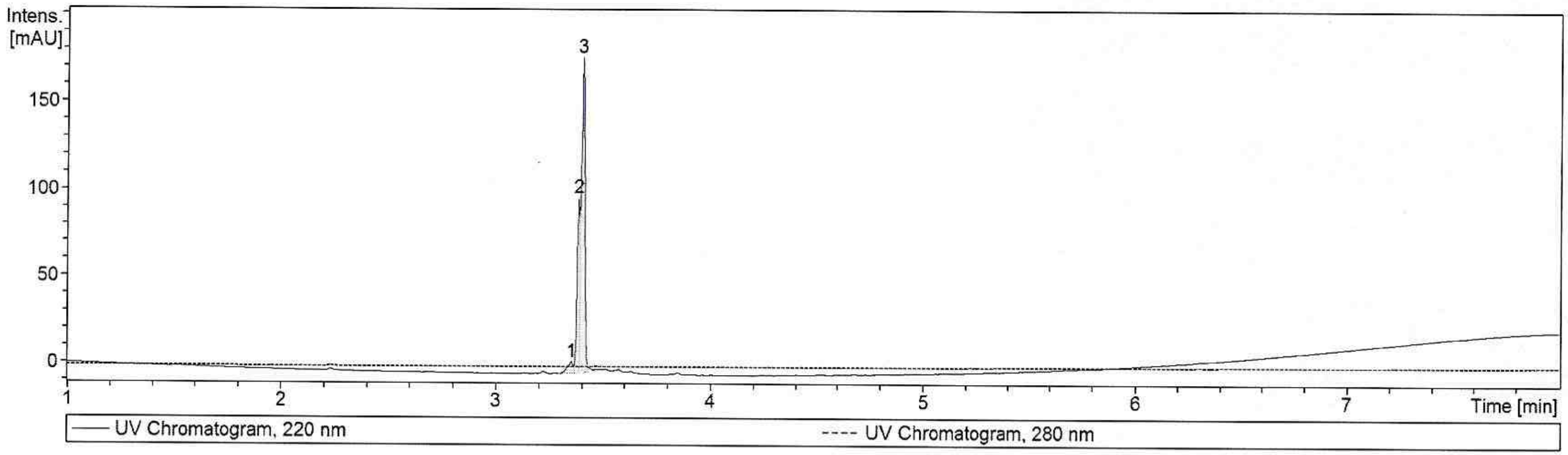

Target Mass	Meas. Mass	Expec. Mass	Delt. Mr [Da]	Intensity	Area	Area Fraction [%]
Cmpd 3; 3.40 min; Pep Mr: 1866.13	1866.13	1867.00	-0.87	175	207	67.2

#	RT [min]	Area	Area Frac. %
1	3.35	12.524	4.06
2	3.39	88.691	28.74
3	3.40	207.370	67.20

Peptide QC Report LB1429 16-34

Cmpd 2, 3.39 min

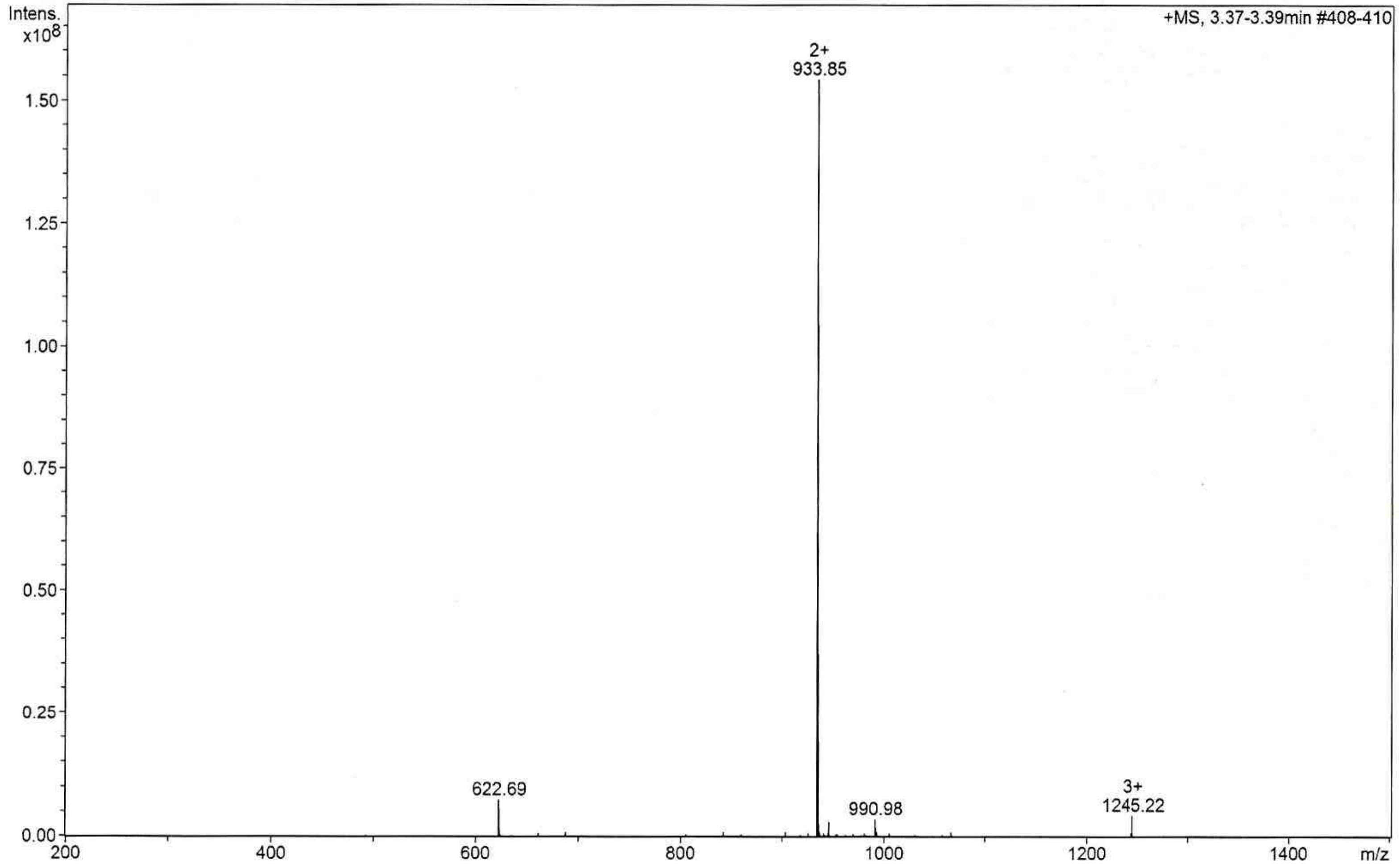

new england peptide

Peptide QC Report LB1429 16-34

Cmpd 3; 3.40 min; Pep Mr: 1866.13

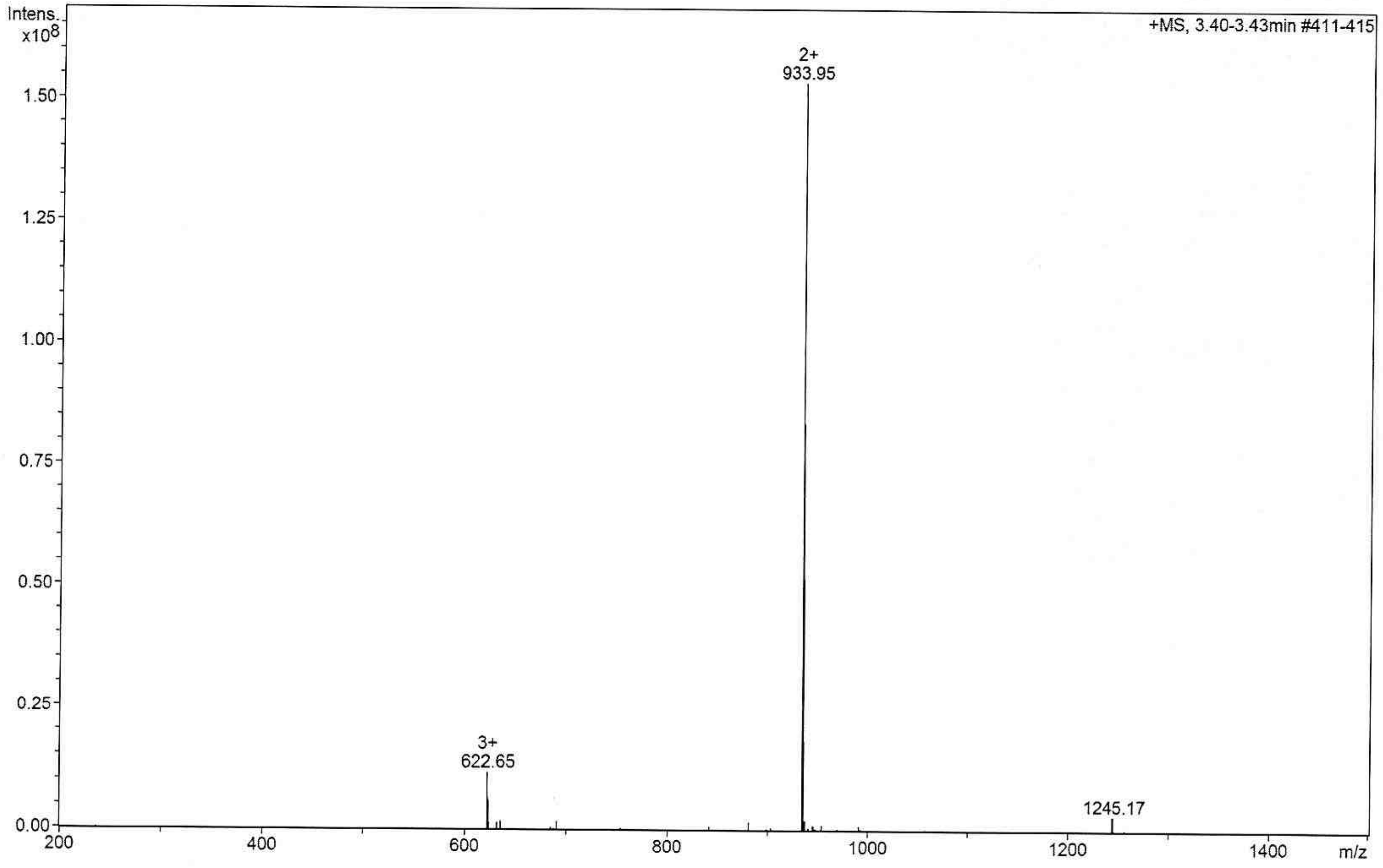

Supplement: Supplementary file 11 [file DataSheet12.PDF]
