# Supplementary material for: Screening of Hydrocarbon-Stapled Peptides for Inhibition of Calcium-Triggered Exocytosis
Source: Front Pharmacol. 2022 Jun 17;13:891041. doi: 10.3389/fphar.2022.891041 (PMC9258623; doi:10.3389/fphar.2022.891041)

new england peptide™

Certificate of Analysis

**Sequence:** [Cyc(4,11)]Ac-SKD(R8)GIRTLV(S5)LDEQGEQL-amide		
**Peptide Name:**		**Date:** 8/1/2017
**Order#:** P611359	**Lot#:** LB1364	**Amount:** 5.1mg

Quality Control Specifications:

QC Test	QC Specifications	Results
Purity by HPLC	≥**90%** by percent area	**Pass**
Mass Identification by Mass Spectral Analysis	Calculated Mass within 0.1% of Molecular Weight: **2235**	**Pass**
Concentration/ Net Peptide	Amino Acid Analysis (AAA) determining original concentration/net peptide content.	**N/A**

Product: Research Grade Custom Peptide containing traces of Trifluoroacetate (TFA) salts.

Formulation:
Final concentration: N/A
Final form: Dry

Stability and Conditions: Refer to the Quality Control Detail Information on our website at www.newenglandpeptide.com/support/quality-control-information . As always, NEP has individual batch records stored electronically for each peptide that includes traceable lot numbers of raw materials used during synthesis. Should you require this information, email sales@newenglandpeptide.com with your peptide lot number.

Notes (if applicable):

AD
Approval/Initials

For Science... From Science.

New England Peptide Inc., 65 Zub Lane, Gardner, MA 01440 ▪ **Phone** 888-343-5974 ▪ **Fax** 978-630-0021

www.NewEnglandPeptide.com

Peptide QC Report LB1364 12-15

Analysis Name	D:\Data\LB1364 12-15_141922_P1-A-4_01_71062.D
Sample Name	LB1364 12-15
Method	APRIL20171.2mLperMIN_NEPO AHIGH_71062.m
Instrument	amaZon SL

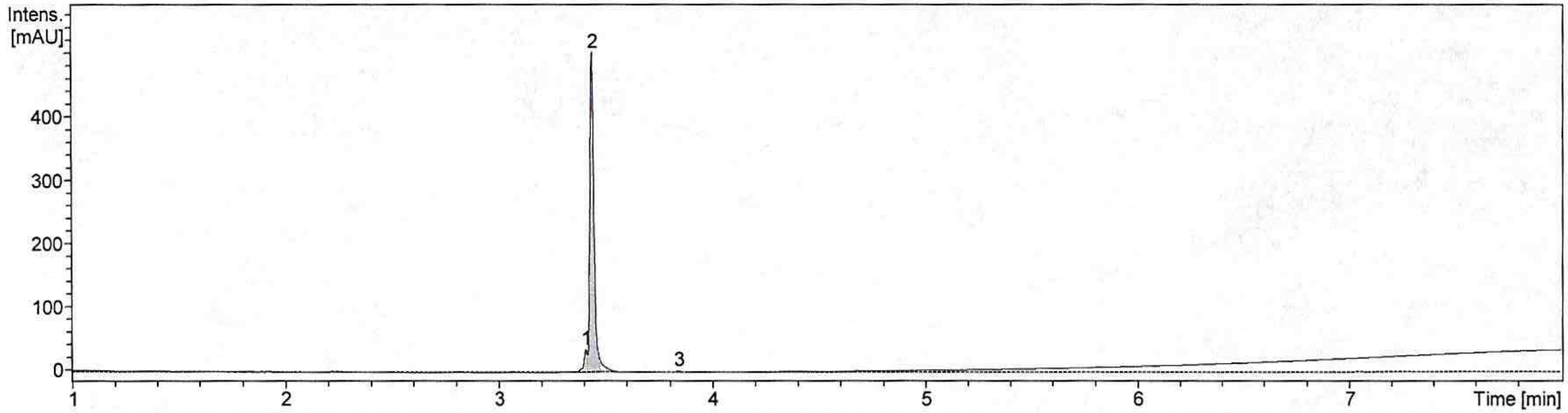

— UV Chromatogram, 220 nm ---- UV Chromatogram, 280 nm

Target Mass	Meas. Mass	Expec. Mass	Delt. Mr [Da]	Intensity	Area	Area Fraction [%]
Cmpd 2; 3.44 min; Pep Mr: 2234.25	2234.25	2235.00	-0.75	498	632	93.3

#	RT [min]	Area	Area Frac. %
1	3.41	41.8613	6.18
2	3.44	632.2511	93.27
3	3.84	3.7519	0.55

Cmpd 2; 3.44 min; Pep Mr: 2234.25

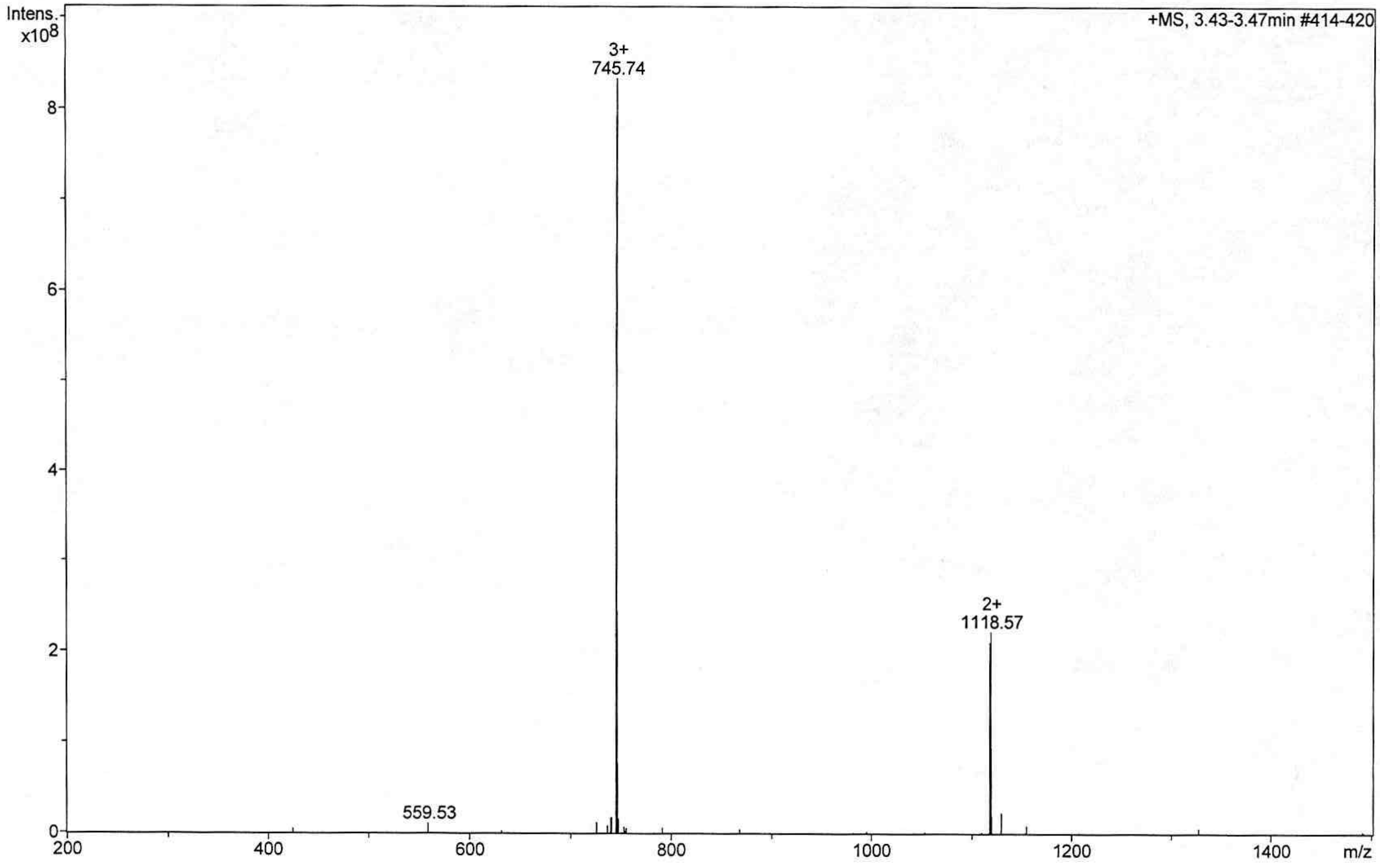

Supplement: Supplementary file 12 [file DataSheet8.PDF]
