# Supplementary material for: Screening of Hydrocarbon-Stapled Peptides for Inhibition of Calcium-Triggered Exocytosis
Source: Front Pharmacol. 2022 Jun 17;13:891041. doi: 10.3389/fphar.2022.891041 (PMC9258623; doi:10.3389/fphar.2022.891041)

new england peptide™

Certificate of Analysis

**Sequence:** [Cyc(4,8;11,15)]Ac-SKD(S5)GIR(S5)LV(S5)LDE(S5)GEQL-amide		
**Peptide Name:**		**Date:** 8/7/2017
**Order#:** P611359	**Lot#:** LB1542	**Amount:** 5.1mg

Quality Control Specifications:

QC Test	QC Specifications	Results
Purity by HPLC	**≥90%** by percent area	**Pass**
Mass Identification by Mass Spectral Analysis	Calculated Mass within 0.1% of Molecular Weight: **2215**	**Pass**
Concentration/ Net Peptide	Amino Acid Analysis (AAA) determining original concentration/net peptide content.	**N/A**

Product: Research Grade Custom Peptide containing traces of Trifluoroacetate (TFA) salts.

Formulation:
Final concentration: N/A
Final form: Dry

Stability and Conditions: Refer to the Quality Control Detail Information on our website at www.newenglandpeptide.com/support/quality-control-information . As always, NEP has individual batch records stored electronically for each peptide that includes traceable lot numbers of raw materials used during synthesis. Should you require this information, email sales@newenglandpeptide.com with your peptide lot number.

Notes (if applicable):

Approval/Initials

For Science... From Science.

New England Peptide Inc., 65 Zub Lane, Gardner, MA 01440 ■ **Phone** 888-343-5974 ■ **Fax** 978-630-0021

www.NewEnglandPeptide.com

new england peptide

Peptide QC Report LB1542 5-24

Analysis Name	D:\Data\LB1542 5-24_143061_P1-D-8_01_71578.D
Sample Name	LB1542 5-24
Method	APRIL20171.2mLperMIN_NEPO AHIGH_71578.m
Instrument	amaZon SL

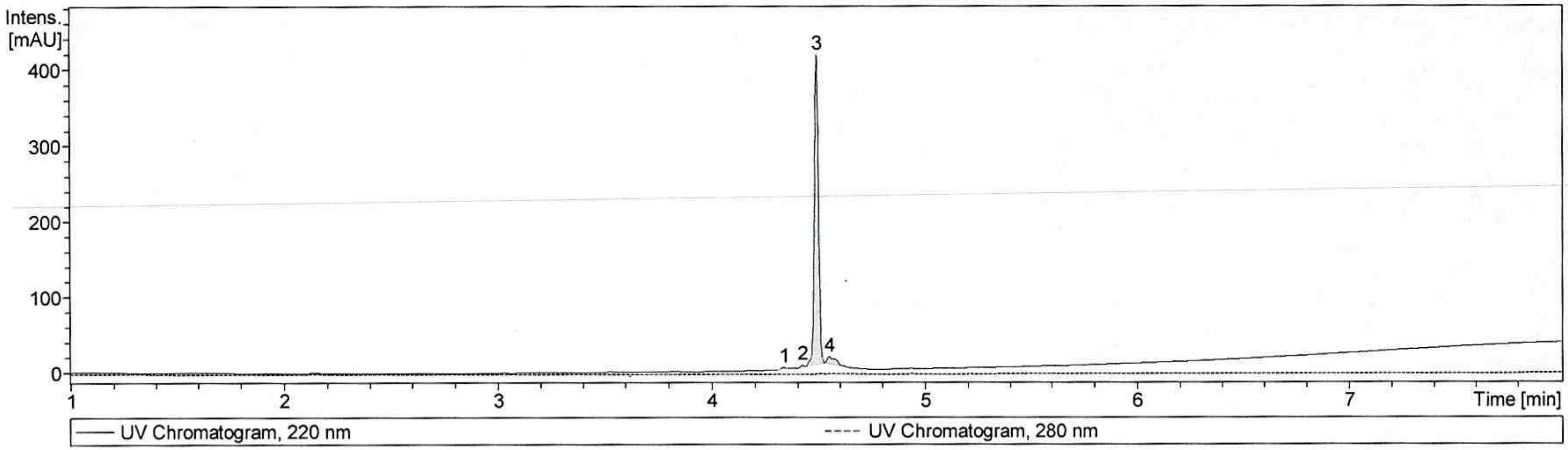

Target Mass	Meas. Mass	Expec. Mass	Delt. Mr [Da]	Intensity	Area	Area Fraction [%]
Cmpd 3; 4.49 min; Pep Mr: 2214.15	2214.15	2215.00	-0.85	418	545	94.3

#	RT [min]	Area	Area Frac. %
1	4.33	2.7407	0.47
2	4.42	2.8883	0.50
3	4.49	544.5035	94.29
4	4.55	27.3173	4.73

Peptide QC Report LB1542 5-24

Cmpd 3; 4.49 min; Pep Mr: 2214.15

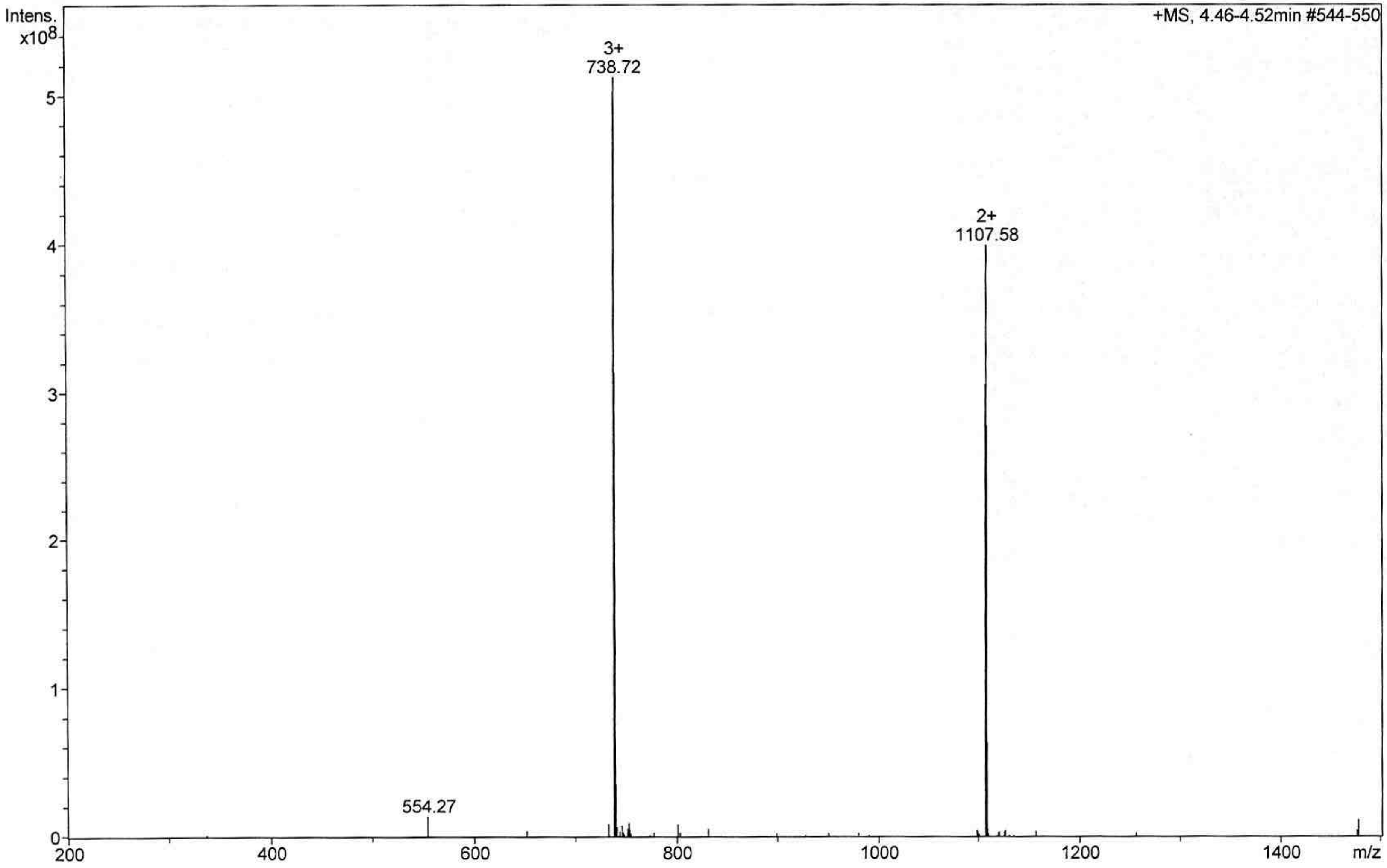

Supplement: Supplementary file 13 [file DataSheet10.PDF]
